# SCORT–Cas13d Nanotherapy Precisely Targets the ‘Undruggable’ Transcription Factor HoxB13 in Metastatic Prostate Cancer In Vivo

**DOI:** 10.1002/advs.202417605

**Published:** 2025-05-11

**Authors:** Zhifen Cui, Furong Huang, Kun Fang, Jingyue Yan, Yuebao Zhang, Diana D. Kang, Yufan Zhou, Yue Zhao, Jeffrey I. Everitt, William Hankey, Andrew J Armstrong, Jiaoti Huang, Hongyan Wang, Victor X. Jin, Yizhou Dong, Qianben Wang

**Affiliations:** ^1^ Department of Pathology Duke University School of Medicine Durham NC 27710 USA; ^2^ Medical College of Wisconsin Cancer Center Medical College of Wisconsin 8701 Watertown Plank Road Milwaukee WI 53226 USA; ^3^ Division of Pharmaceutics & Pharmacology College of Pharmacy The Ohio State University Columbus OH 43210 USA; ^4^ Icahn Genomics Institute Precision Immunology Institute Department of Immunology and Immunotherapy Department of Oncological Sciences Tisch Cancer Institute Biomedical Engineering and Imaging Institute Friedman Brain Institute Icahn School of Medicine at Mount Sinai New York NY 10029 USA; ^5^ Department of Biochemistry and Structural Biology University of Texas Health San Antonio San Antonio TX 78229 USA; ^6^ Department of Medicine Duke University School of Medicine Durham NC 27710 USA; ^7^ Duke Cancer Institute Center for Prostate and Urologic Cancer Durham NC 27710 USA; ^8^ Department of Cell Biology Duke University School of Medicine Durham NC 27710 USA

**Keywords:** HoxB13, mechanistic insights into therapeutic action, metastatic prostate cancer, SCORT‐Cas13d nanotherapy, SCORT nanoparticles, undruggable oncogenic transcription factors

## Abstract

Metastatic cancer, the primary cause of cancer mortality, frequently exhibits heightened dependence on certain transcription factors (TFs), which serve as master regulators of oncogenic signaling yet are often untargetable by small molecules. Selective Cell in ORgan Targeting (SCORT) nanoparticles are developed for precise *CRISPR/Cas13d* mRNA and gRNA delivery to metastatic cancer cells in vivo, aiming to knock down the undruggable oncogenic TF HoxB13. In prostate cancer liver metastasis models driven by HoxB13, repeated systemic SCORT‐*Cas13d*‐g*HoxB13* treatment significantly decreases HoxB13 expression, reduces metastasis, and extends mouse survival. Prolonged treatment shows no significant impact on major organ function, histology or immune markers. Mechanistically, SCORT‐*Cas13d*‐g*HoxB13* treatment suppresses metastatic tumor proliferation and angiogenesis while promoting apoptosis by regulating multiple gene pathways. Unexpectedly, it inhibits the non‐canonical, EMT‐independent oncogenic function of Snail. These findings suggest that SCORT‐*Cas13d*‐g*HoxB13* can effectively and safely target the undruggable HoxB13 in metastatic prostate cancer, positioning CRISPR/Cas13d as a potential treatment.

## Graphical abstract

SCORT–*Cas13d*–pre‐g*HoxB13* lipid nanoparticles precisely deliver *Cas13d* mRNA and guide RNAs to metastatic prostate tumors in the liver, knocking down the “undruggable” transcription factor HoxB13. This approach suppresses metastatic tumor growth, extends mouse survival, and does not induce major organ toxicity or immune activation, offering a safe and effective gene therapy platform for advanced prostate cancer.

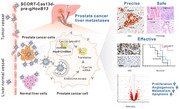

## 1 Introduction

Metastatic cancer remains the leading cause of cancer mortality, accounting for most cancer‐related deaths worldwide.^[^ ^1^ ^]^ Prostate cancer is the second most frequently diagnosed cancer in men worldwide, with an estimated 1.4 million new cases and over 375 000 deaths globally in 2020, the majority of which are attributed to metastatic disease.^[^ ^2^ ^]^ This profound clinical impact underscores the urgent need to understand and target the molecular drivers of metastatic spread.^[^ ^3^ ^]^Among these drivers, transcription factors (TFs) arguably play a more critical role than conventional signaling oncoproteins, as TFs serve as keystones in the deregulated signaling pathways and are the master regulators of many signaling proteins—a concept suggested by James E. Darnell over 20 years ago.^[^ ^4^ ^]^ Indeed, cancer cells frequently develop a marked dependency on certain TFs, a phenomenon known as ‘transcriptional addiction’ in cancer.^[^ ^5^ ^]^ Unfortunately, apart from nuclear hormone receptors, most TFs are considered ‘undruggable’ due to conformational variability and the lack of distinct small‐molecule binding sites.^[^ ^6^ ^]^

Several innovative protein‐targeting strategies have emerged to target these traditionally ‘undruggable’ TFs. However, many of these strategies are indirect, employing kinase inhibitors to modulate TF function, epigenetic inhibitors to alter TF‐bound chromatin, and agents that disrupt TF‐cofactor interactions.^[^ ^6^, ^7^ ^]^ Proteolysis targeting chimeras (PROTACs) are heterobifunctional small molecules designed to bind to TFs and recruit endogenous E3 ubiquitin ligases for the targeted degradation of TFs. Yet, the efficacy of these molecules hinges on the presence of accessible binding sites, which are often lacking in classically ‘undruggable’ TFs.^[^ ^6^, ^7^, ^8^ ^]^ As a result, current PROTACs largely depend on pre‐existing ligands for proteins already classified as druggable, such as the androgen receptor (AR) and estrogen receptor (ER). Furthermore, not all ubiquitin E3 ligases are effective in the degradation of certain proteins.^[^ ^6^, ^7^, ^8^ ^]^ Nucleic acid‐based therapies have emerged as crucial alternative strategies with the potential to directly target traditionally undruggable genes, including oncogenic TFs. However, antisense oligonucleotides (ASOs) have primarily been utilized in the treatment of neurological disorders, and none of the approved ASOs target undruggable proteins directly.^[^ ^8^ ^]^ Although RNA interference (RNAi) is promising, clinical trials have revealed that certain siRNA drugs targeting TFs, such as DCR‐Myc, showed limited knockdown efficacy.^[^ ^8^ ^]^ Moreover, RNAi‐based approaches may silence target genes but can also produce significant off‐target effects due to partial sequence complementarity and the roles of RNAi machinery in endogenous processes.^[^ ^9^ ^]^ CRISPR/Cas9‐based therapy, which is currently used mainly ex vivo for non‐cancer diseases, demonstrates higher efficiency than other nucleic‐acid‐based approaches; however, it also carries potential risks for irreversible genomic alterations and immunogenicity issues due to preexisting immunity to Cas9 in humans.^[^ ^8^, ^10^ ^]^ Hence, there is a pressing need to develop novel approaches that can conveniently, effectively, and specifically target ‘undruggable’ oncogenic TFs.

We therefore employed the RNA‐targeting CRISPR/Cas13d system for the knockdown of TF mRNAs in metastatic cancer. The Cas13d enzyme^[^ ^11^ ^]^ is a compact and efficient mediator of RNA‐targeting gene therapy, capable of degrading mRNA without causing permanent (on‐ or off‐target) DNA alterations associated with CRISPR/Cas9.^[^ ^12^ ^]^Compared to RNAi, Cas13d typically exhibits fewer off‐target transcriptomic effects,^[^ ^9^ ^]^ and our group and others have shown that CasRx (the Cas13d (NLS) protein from the *Ruminococcus flavefaciens* strain XPD3002) can achieve high‐efficiency knockdown in vitro and in vivo with minimal off‐target toxicity.^[^ ^11^, ^13^ ^]^ By simply changing guide RNAs (gRNAs), CasRx can be readily adapted to target diverse oncogenic TFs, presenting a versatile strategy for tackling metastatic cancers.

Although CasRx facilitates potent and specific gene knockdown, the efficient and targeted delivery of CasRx—preferably in its mRNA format, due to rapid gene‐editing kinetics and reduced off‐target effects compared to plasmid DNA^[^ ^13^, ^14^ ^]^—alongside gRNA to metastatic cancer cells in vivo remains a considerable challenge. Lipid nanoparticles (LNPs), comprised of ionizable lipid, cholesterol, phospholipid, and polyethylene glycol (PEG)‐lipid, have been extensively investigated for mRNA delivery in various biomedical applications.^[^ ^15^ ^]^ Notably, LNPs administered intravenously tend to accumulate primarily in the liver, a property that could be beneficial for treating liver metastases^[^ ^16^ ^]^—a deadly progression of several cancers, including colorectal and prostate cancer. Nevertheless, clearance primarily by the reticuloendothelial system (RES), including liver‐resident phagocytes such as Kupffer cells, and additional uptake by hepatocytes pose challenges when aiming to direct these nanoparticles to metastatic tumor cells.^[^ ^15^, ^17^ ^]^ Therefore, precise LNP design is imperative to ensure that systemically delivered LNP‐mRNA therapeutics effectively reach metastatic cancer cells in the liver, thereby achieving potent anti‐tumor effects.

In this study, we introduced a strategy termed Selective Cell in ORgan Targeting (SCORT) that enables systematic engineering of nanoparticles for precise delivery of RNA cargoes, including reporter mRNA, *CasRx* mRNA, and gRNA oligonucleotides, to metastatic cancer cells in the mouse liver following I.V. administration. We used metastatic castration‐resistant prostate cancer (CRPC) liver metastasis as our experimental model since it is present in ≈25% of patients with advanced disease, according to autopsy studies.^[^ ^18^ ^]^ It is associated with the worst prognosis and poor response to hormonal therapy, taxane chemotherapy, and prostate‐specific membrane antigen (PSMA)‐directed radioligand therapy; thus, overcoming CRPC liver metastases represents a major unmet medical need.^[^ ^19^ ^]^ Metastatic CRPC highly expresses the undruggable oncogenic TF HoxB13,^[^ ^20^ ^]^ a member of the 39 human members of the Hox subgroup within the homeobox family of TFs.^[^ ^21^ ^]^ We demonstrated that SCORT LNPs, which carry *CasRx* mRNA and unprocessed gRNA (pre‐gRNAs) targeting *HoxB13* (designated as SCORT‐*CasRx*‐pre‐g*HoxB13*) decreased HoxB13 expression in the metastatic tumors, inhibited metastasis, and prolonged survival of mice bearing AR‐positive (AR+) or negative (AR‐) CRPC tumors by inhibiting cell proliferation, angiogenesis, and metabolism, and inducing apoptosis. Using SCORT‐*CasRx*‐pre‐g*HoxB13* as a tool, we also unexpectedly found that HoxB13 knockdown inhibited the non‐canonical, epithelial‐mesenchymal transition (EMT)‐independent oncogenic function of Snail. Notably, prolonged administration of SCORT‐*CasRx*‐pre‐g*HoxB13* did not significantly alter body weight, hepatic and renal function, or levels of chemokines and cytokines, effectively highlighting its safety. This research represents a pioneering effort to precisely, effectively, and safely target undruggable oncogenic TFs via nanoparticle‐delivered CasRx RNA‐targeting gene therapy, and to use CRISPR as a treatment for metastatic cancers. The SCORT‐*Cas13d*‐g*TF* system, a highly flexible technology, has the potential to significantly impact translational and basic sciences by targeting and studying previously undruggable oncogenic TFs in metastatic prostate cancer and other solid tumors, using various gRNAs and modifications of nanomaterials.

## 2 Results

### 2.1 Specific and Effective Targeting of HoxB13 by CasRx Inhibits CRPC Cell Proliferation and Invasion

**Figure 1:**
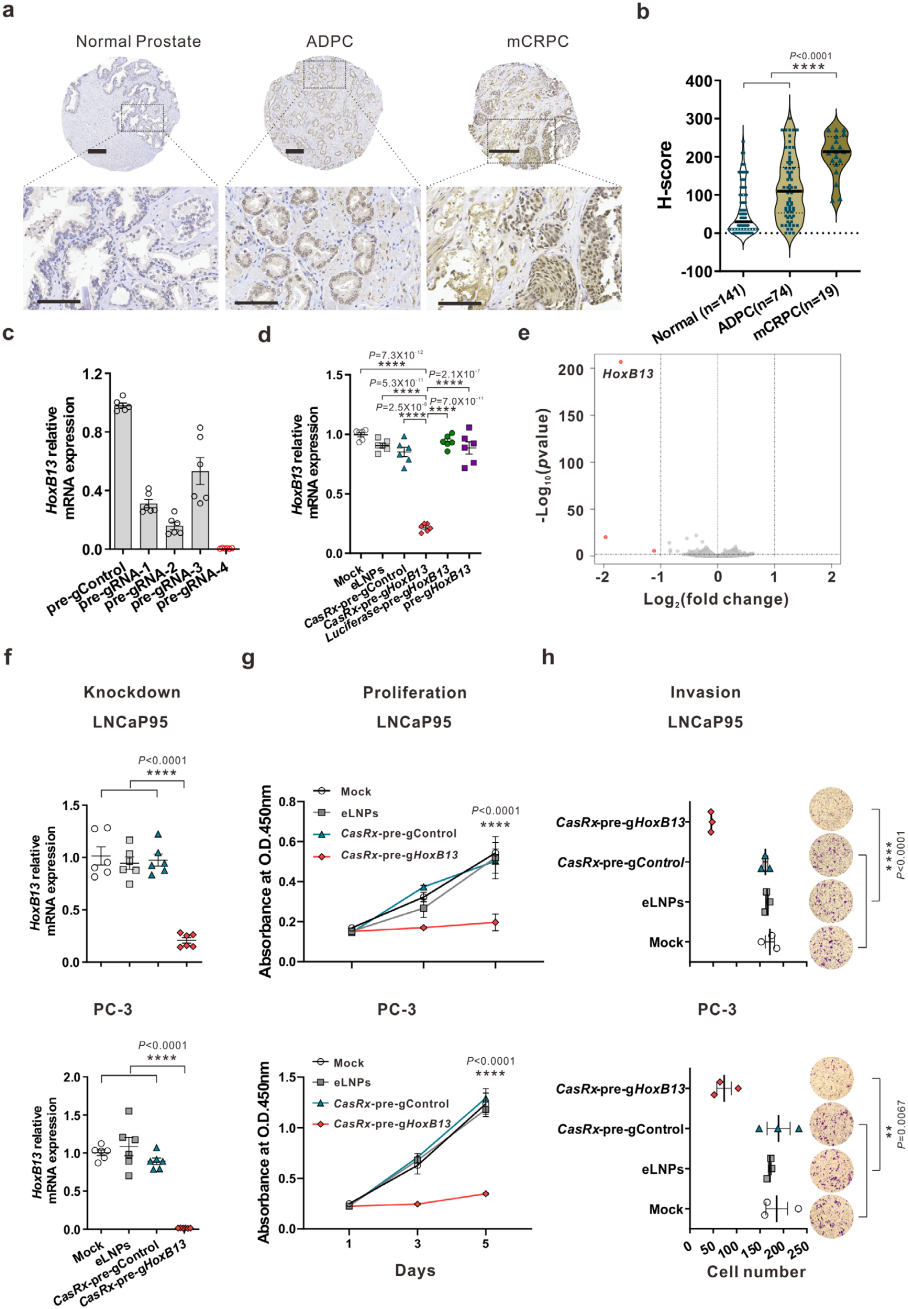
CasRx‐mediated specific HoxB13 knockdown inhibits proliferation and invasion of both AR+ and AR‐ CRPC cells. a) Representative HoxB13 immunoreactivity in normal human prostate, ADPC, and mCRPC tissues. Scale bar: 200 µm (upper panels);100 µm (lower panels). b) H‐score of HoxB13 nuclear staining in human tissues. The bar shows the median of each group, while each dot represents an individual sample. *P‐*values were calculated by One‐way ANOVA. ^****^ *p* < 0.0001. c) *HoxB13* mRNA level in 293FT cells after transfection with CasRx plasmid and different pre‐gRNAs designed to target *HoxB13*. *HoxB13* transcript levels are normalized by 1*8s‐rRNA*. Data are presented as mean ± SEM with *n* = 6 biologically independent replicates. d) *HoxB13* mRNA level in 293FT cells after transfection with *CasRx* or *luciferase* mRNA and pre‐gControl or pre‐g*HoxB13*. Data are presented as mean ± SEM with n = 6 biologically independent replicates. *P‐*values were calculated by a two‐tailed Student's *t‐*test. ^****^ *p* < 0.0001. e) Volcano plot of gene expression changes between *CasRx*‐pre‐g*HoxB13* and *CasRx*‐pre‐gControl transfected 293FT cells for 24 h. RNA sequencing was performed in biological triplicates. Significantly differentially expressed genes (Fold change>2, *q*‐value<0.01) are shown in red. f‐h) LNCaP95 (upper), and PC‐3 cells (lower) were incubated with mock, empty LNPs (eLNPs), LNPs encapsulating *CasRx* mRNA and pre‐gControl or pre‐g*HoxB13*, respectively, and subjected for analysis. HoxB13 transcript levels. Data are presented as mean ± SEM with n = 5 biologically independent replicates (f). Cell proliferation. Data are presented as mean ± SD with n = 5 biologically independent replicates (g). Cell invasion. The number of invaded cells was quantified (left), and representative images are shown on the right (100x magnification). Data are presented as mean ± SEM of three representative fields from one of three biologically independent experiments (h). *P*‐values were calculated by One‐way ANOVA. ^**^ *p* < 0.01, ^****^ *p* < 0.0001.

Our previous studies have found that silencing HoxB13 with RNAi significantly decreases CRPC cell growth in vitro and in xenograft models.^[^ ^20a^ ^]^ To examine the clinical relevance of HoxB13, we conducted immunohistochemical (IHC) analysis on normal human prostate tissues, earlier androgen‐dependent prostate cancer (ADPC) patient tissues, and metastatic CRPC patient tissues. CRPC samples exhibited significantly higher HoxB13 staining than ADPC or normal prostate samples (**Figure** **1**a,b), suggesting that HoxB13 expression increases during prostate cancer progression to the lethal phase. To knock down HoxB13 using CasRx, we screened a series of pre‐gRNAs to identify one (pre‐g*HoxB13*‐4) that mediates the most potent knockdown of *HoxB13* (∼99%) mRNA in human 293FT cells (Figure 1c). Furthermore, we found that transfection with pre‐g*HoxB13*‐4 gRNA alone or *luciferase* mRNA, in the absence of *CasRx* mRNA, did not lead to a reduction in *HoxB13* mRNA levels (Figure 1d), which is consistent with the notion that Cas13 guide RNAs have no RNA interference effects.^[^ ^22^ ^]^ We next evaluated the off‐target effects of CasRx‐mediated *HoxB13* gene knockdown using RNA‐seq analysis in 293FT cells. In cells transiently transfected with the CasRx‐pre‐gHoxB13 vector, we observed significant changes only in the targeted gene, i.e., *HoxB13*, and two other genes, *FACMR* and *CERS3* (Figure 1e; Figure S1a, Supporting Information), which were previously reported to be direct target genes of HoxB13 in other systems.^[^ ^23^ ^]^ Our findings of no significant off‐target effects resulting from CasRx‐gHoxB13 targeting are consistent with previous reports of the high specificity of CasRx‐mediated RNA knockdown in mammalian cells.^[^ ^11^, ^13^ ^]^ Finally, we examined the effects of CasRx‐mediated HoxB13 knockdown on the CRPC cellular phenotypes. We chose two CRPC models, LNCaP95 and PC‐3, to cover the spectrum of CRPC patients: LNCaP95 represents the majority who are AR‐positive, while PC‐3 accounts for the significant AR‐negative minority (≈30%).^[^ ^24^ ^]^ Significantly, transfection with *CasRx* mRNA and pre‐g*HoxB13* oligos reduced HoxB13 mRNA and protein expression in LNCaP95 and PC‐3 cells (Figure 1f; Figure S1b,c, S13, Supporting Information), concurrently inhibited the growth (Figure 1g) and invasion (Figure 1h) of these CRPC cells. In contrast, *CasRx*‐pre‐g*HoxB13* did not affect the proliferation of the mouse hepatocyte cell line AML12 or the immortalized human liver epithelial cell line THLE2 (Figure S1d, Supporting Information), suggesting that *CasRx*‐pre‐g*HoxB13* is not cytotoxic. Collectively, these data demonstrate that CasRx‐mediated, HoxB13‐specific knockdown effectively suppresses CRPC cell growth and invasion in vitro without causing cytotoxicity.

### 2.2 Development of the SCORT LNP System for Effective and Precise mRNA Delivery to Target Liver Metastatic Cancer Cells

We next aimed to develop an LNP system to effectively and precisely deliver *CasRx* mRNA/pre‐gRNA to target HoxB13 within metastatic CRPC cells in the liver. We developed the SCORT LNP‐mRNA delivery approach, which synergistically combines the unique characteristics of nanomaterials with the high transferrin receptor 1 (TfR 1) expression in CRPC cells—recognized by the E3 aptamer^[^ ^25^ ^]^—and the fenestrated vasculature characteristic of liver metastases. Our strategy includes: (1) utilizing our recently identified ionizable lipid FTT5^[^ ^15b^ ^]^ as the main lipid for efficient in vivo delivery of long mRNA, specifically the *CasRx* mRNA (2.9 kb); (2) incorporating active targeting through the addition of the E3 aptamer into LNP, an RNA aptamer selected through a process targeting cells^[^cell‐ internalization SELEX (Systematic Evolution of Ligands by EXponential enrichment)] rather than proteins.^[^ ^25a^ ^]^ This aptamer has initially demonstrated internalization into ADPC and CRPC cells but not normal prostate cells, and subsequently exhibited broad, specific targeting capabilities across multiple cancer types through binding to TfR 1^[^ ^25^, ^26^ ^]^; (3) fine‐tuning the molar ratios of PEG‐lipids to enhance the preferential delivery of LNP to CRPC cells over hepatocytes. These ratios affect nanoparticle diameters, which are crucial for the delivery of LNP to various cell types.^[^ ^27^ ^]^ It is noteworthy that smaller LNP particles navigate through the fenestrations, which are ≈100–140 nm in diameter,^[^ ^27^, ^28^ ^]^ between liver sinusoidal endothelial cells (LSECs) to reach hepatocytes. Additionally, the vasculature of tumors often features an incomplete endothelial lining, presenting relatively larger pores compared to those in most normal microvessels.^[^ ^29^ ^]^

**Figure 2:**
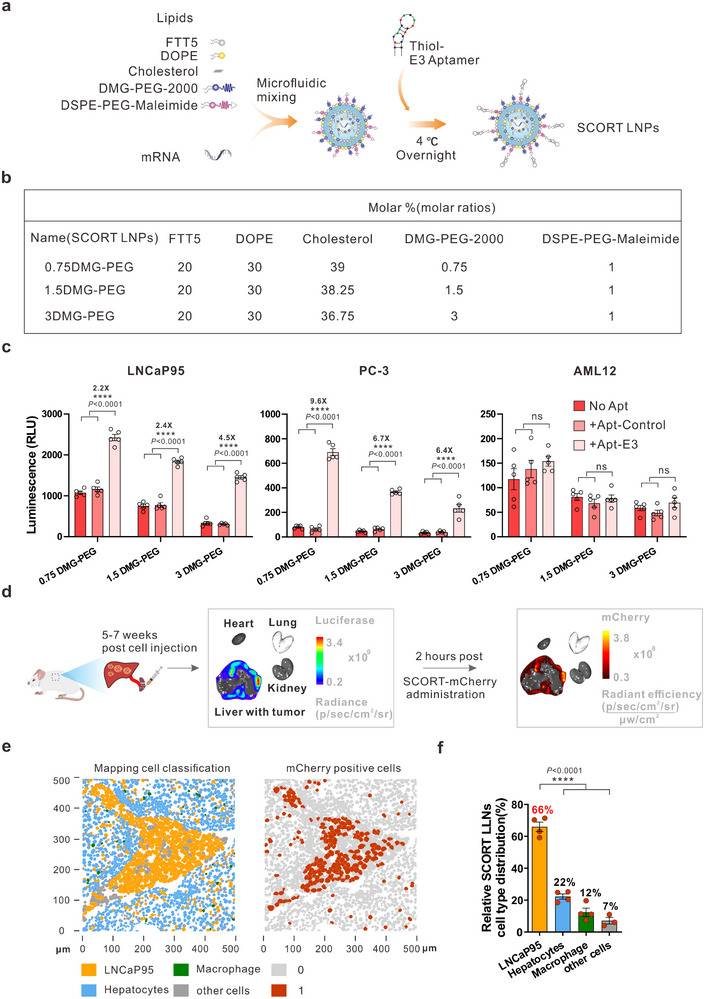
Construction of SCORT LNPs for specific targeting of CRPC cells in vitro and in vivo. a) Schematic of SCORT LNPs generation through microfluidic mixing and E3 aptamer conjugation. b) Composition of LNP candidates. c) Bioluminescence signal in LNCaP95, PC‐3, and AML12 cells after 24 h incubation with *luciferase* mRNA‐encapsulated LNP candidates modified with no aptamer (No Apt), a control aptamer (+Apt‐Control), or E3 aptamer (+Apt‐E3). The experiment was conducted with *n* = 5 biologically independent replicates. Data are presented as mean ± SEM. *P*‐value was calculated by One‐way ANOVA. ^****^ *p* < 0.0001, ns, not significant. D) Schematic of the establishment of LNCaP95 liver metastasis model and distribution of *mCherry* mRNA‐encapsulated LNPs in major organs. e) Representative images of cell classification mapping (left panel) and mCherry‐positive cells (right panel) by IMC analysis. f) Percentage of mCherry‐positive cells in different cell types. Each red point represents the distribution in an individual mouse (*n* = 4, one batch of SCORT‐mCherry per mouse). Data are presented as mean ± SEM. *P* value was calculated by One‐way ANOVA. ^****^ *p* < 0.0001.

In adherence to these guiding principles, we first developed three modified FTT5 LNPs (mFTT5 LNPs), each comprising five components (**Figure** **2**a): four basic elements, which are FTT5, DOPE (helper lipid), cholesterol, and DMG‐PEG 2000 (at molar ratios of 0.75, 1.5, or 3), along with a fifth molecule, DSPE‐PEG‐maleimide, for E3 aptamer coupling (Figure 2b). These mFTT5 LNPs were then loaded with the cargo mRNA using the NanoAssembler microfluidic mixing device to attain a high mRNA encapsulation rate while reducing batch‐to‐batch variability. Following this, E3‐modified mFTT5 LNPs (SCORT LNPs) were obtained through a thiol‐maleimide cross‐linking reaction (Figure 2a,b). Physicochemical characterization of these LNPs found that the mFTT5 with a 0.75 ratio of DMG‐PEG2000 had the highest encapsulation rate, reaching almost 90% (Figure S2a, Supporting Information). Transmission electron microscopy (TEM) revealed that both mFTT5 LNPs and SCORT LNPs displayed spherical structures (Figure S2b, Supporting Information). Interestingly, decreasing the molar ratio of DMG‐PEG2000 led to an increase in mean particle size, expanding from 118.0 to 161.5 nm for mFTT5 LNPs and from 129.5 to 179.3 nm for SCORT LNPs, accompanied by a noticeable decrease in polydispersity index (PDI) (Figure S2c, Supporting Information). This trend is consistent with the role of PEG‐DMG in providing steric stabilization; insufficient coverage may promote partial aggregation.^[^ ^30^ ^]^ Notably, all formulations maintained PDI values below 0.2 or very close to it, indicating highly monodisperse characteristics^[^ ^31^ ^]^ (Figure S2c, Supporting Information).

We examined the delivery efficiencies of three mFTT5 LNPs and three SCORT LNPs, each carrying *luciferase* mRNA, in LNCaP95 and PC‐3 CRPC cells in vitro. The mouse hepatocyte cell line AML12 served as a control. Incubation of these cells with the mFTT5 formulations carrying *luciferase* mRNA revealed the highest delivery efficiency in LNCaP95 cells, surpassing both PC‐3 and AML12 cells (Figure 2c). Significantly, the SCORT LNPs (+Apt‐E3) achieved a substantial increase in luciferase expression compared to the mFTT5 LNPs (No aptamer) or control aptamer‐ modified mFTT5 LNPs (+Apt‐Control), with enhancements of up to 4.5‐fold in LNCaP95 cells and 9.6‐fold in PC‐3 cells (Figure 2c). No increase was observed in AML12 cells, indicating the tumor selectivity of SCORT LNPs (Figure 2c). Bioluminescence imaging further illustrated the pronounced differences between mFTT5 LNPs and SCORT LNPs in LNCaP95 and PC‐3 cells but not AML12 cells (Figure S3a, Supporting Information). Similar outcomes were also observed in flow cytometry assays with two other reporter genes, *EGFP* and *mCherry* (Figure S3b,c, Supporting Information, gating strategy shown in Figure S12, Supporting Information). Based on these findings, we selected SCORT LNPs with the lowest PEG lipid molar ratio (0.75 DMG‐PEG 2000) for subsequent in vivo assays, due to their superior delivery efficacy in vitro and their large particle sizes.

To evaluate the in vivo mRNA delivery selectivity and efficacy of SCORT LNPs, which contains a 0.75 mol ratio of DMG‐PEG2000 for carrying *mCherry* mRNA (hereafter, SCORT‐*mCherry*), to metastatic CRPC cells following systemic administration, we established a CRPC liver metastasis model using LNCaP95 cells that stably express luciferase (LNCaP95‐Luc) via hemi‐spleen injection.^[^ ^32^ ^]^ Conspicuous metastatic lesions developed 5–7 weeks post‐cell injection, at which point we administered SCORT‐*mCherry* via intravenous injection. Two hours later, robust mCherry fluorescence was detected precisely at the sites of luciferase‐expressing LNCaP95 metastatic tumors, indicating that the SCORT LNP system exhibited a 9.17‐ to 14.45‐fold preference for targeting metastatic CRPC in the liver over normal liver, heart, lung and kidney tissues (Figure 2d; Figure S3d, Supporting Information). To quantify the expression levels of mCherry protein in LNCaP95 tumor cells and compare these with liver cell types, especially hepatocytes, we performed histology analysis using an Imaging Mass Cytometry (IMC) assay. After labeling the corresponding cells with specific antibodies (PSMA for LNCaP95, HNF4α for hepatocytes, and F4/80 for macrophages, primarily Kupffer cells), we observed a remarkable accumulation of mCherry protein predominantly in LNCaP95 tumor cells. This accumulation was 3.23‐fold, 5.73‐fold, and 19.45‐fold higher than in hepatocytes, macrophages, and other cells, respectively, indicating that ≈66% of metastatic CRPC cells expressed mCherry (Figure 2e,f). These data demonstrate the effectiveness and precision of our established SCORT LNP system for mRNA delivery to metastatic cancer cells in the liver.

### 2.3 Systemic SCORT‐*CasRx*‐Pre‐g*HoxB13* Treatment Suppresses CRPC Liver Metastasis and Extends Mice Survival

**Figure 3:**
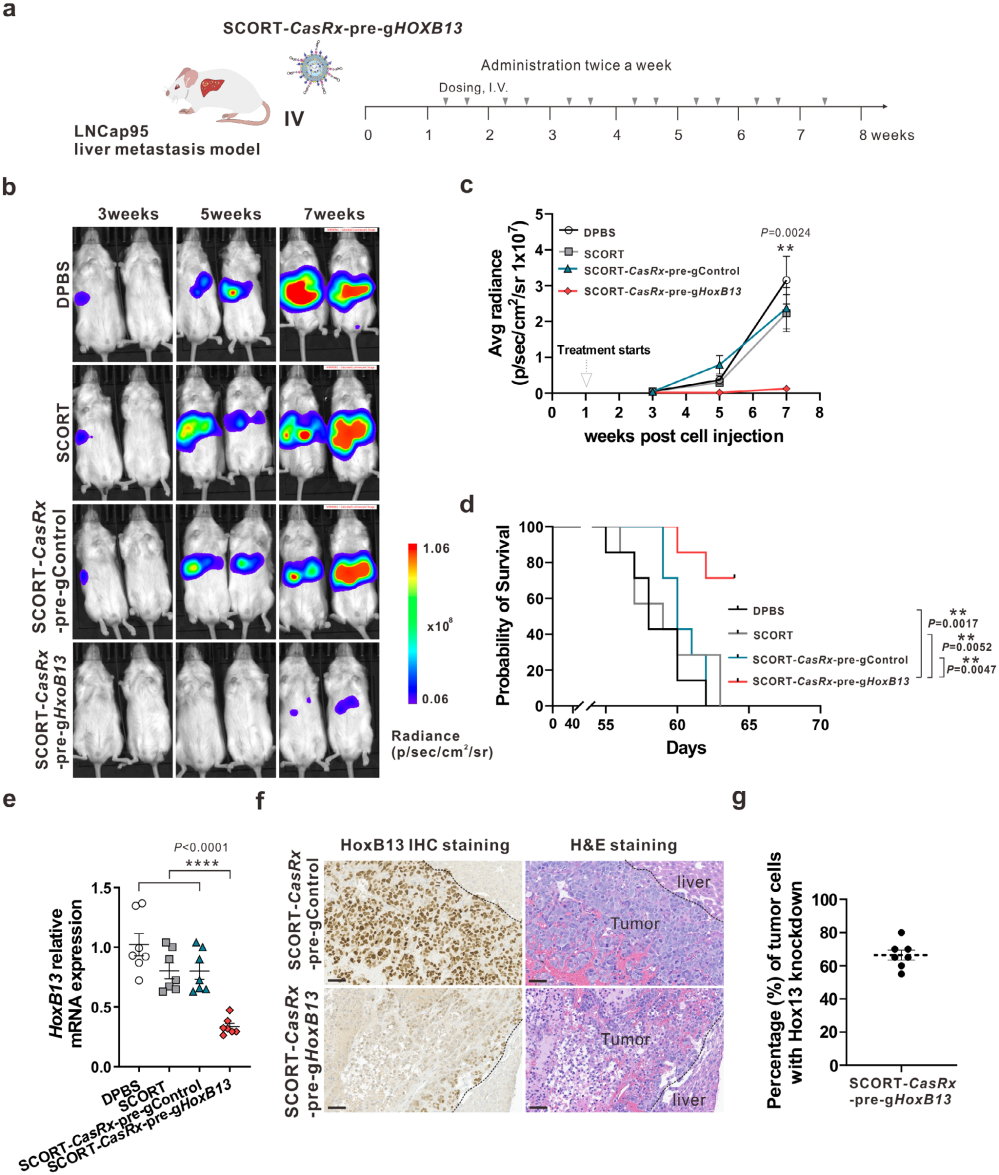
SCORT‐*CasRx*‐pre‐g*HoxB13* treatment suppresses liver metastasis and improves survival in AR+ CRPC liver metastasis mouse model. a) Schematic illustration of the experimental design. The CRPC liver metastasis mouse model was established by hemi‐spleen injection of LNCaP95 cells stably expressing luciferase (LNCaP95‐Luc). One week after cell engraftment, mice received I.V. administration of DPBS, SCORT LNPs, SCORT*‐CasRx*‐pre‐gControl, and SCORT*‐CasRx*‐pre‐g*HoxB13*, receptively, twice a week for 6.5 weeks (*n* = 7). b) Representative bioluminescence imaging of the whole animal on week 3, 5, 7 after cancer cell engraftment. c) Cumulative luciferase counts during the time course. Data are presented as mean ± SEM. *P*‐values were calculated by One‐way ANOVA, ^**^ *p* < 0.01. d) Kaplan‐Meier survival curves. *P* values were determined by the log‐rank (Mantel‐Cox) test. ^**^ *p* < 0.01. e–g) Three days after the last treatments, liver metastatic tumors were subjected to analysis. *HoxB13* transcript levels. Data are presented as mean ± SEM. *P* values were calculated by One‐way ANOVA, ^****^ *p* < 0.0001 (e). Representative images of HoxB13 protein immunostaining and the corresponding H&E staining of the same area. Scale bar: 60 µm. (f). The percentage (%) of tumor cells with HoxB3 knockdown in the SCORT*‐CasRx*‐pre‐g*HoxB13* group. Data is presented as mean ± SEM.(g).

Having established the SCORT LNP‐mRNA delivery system, we next constructed SCORT LNPs carrying *CasRx* mRNA and pre‐g*HoxB13*‐4 oligos (hereafter, SCORT‐*CasRx*‐pre‐g*HoxB13*). To evaluate the therapeutic efficacy of SCORT‐*CasRx*‐pre‐g*HoxB13* in vivo, we utilized the LNCaP95‐Luc CRPC liver metastasis model. Treatments were initiated one week after LNCaP95‐Luc cell injection, with SCORT‐*CasRx*‐pre‐g*HoxB13* administered alongside control groups receiving SCORT LNPs loaded with *CasRx*‐pre‐gControl (SCORT‐*CasRx*‐pre‐gControl), SCORT LNPs alone, and DPBS. Treatments were administered twice weekly until mice in the control group started to succumb, approximately at 6.5 weeks (**Figure** **3**a,d). The administration of SCORT‐*CasRx*‐pre‐g*HoxB13* significantly suppressed metastasis, as evidenced by bioluminescence evaluations at weeks 3, 5, and 7 (Figure 3b,c). Survival analysis consistently underscored the superiority of the treatment, with all mice in the control groups succumbing by ≈9 weeks, while 71% of mice in the SCORT‐*CasRx*‐pre‐g*HoxB13* group remained alive (Figure 3d). As expected, a significant decrease in mRNA (Figure 3e) and protein expression (Figures S4a and S13, Supporting Information) of HoxB13 was noted within the SCORT‐*CasRx*‐pre‐g*HoxB13* group compared to the control groups. Additionally, immunostaining for HoxB13 provided further validation, illustrating notable attenuation in HoxB13 expression within the SCORT‐*CasRx*‐pre‐g*HoxB13* group (Figure 3f; Figure S4b, Supporting Information). To quantify CasRx‐mediated HoxB13 knockdown efficiency at the cellular level, the pathological examination was performed using H&E staining to identify CRPC tumor cells and HoxB13 immunostaining to assess HOXB13 protein expression on the same slide and within the same field. This analysis revealed that ≈66% of tumor cells exhibited a loss of HOXB13 expression upon treatment (Figure 3g).

**Figure 4:**
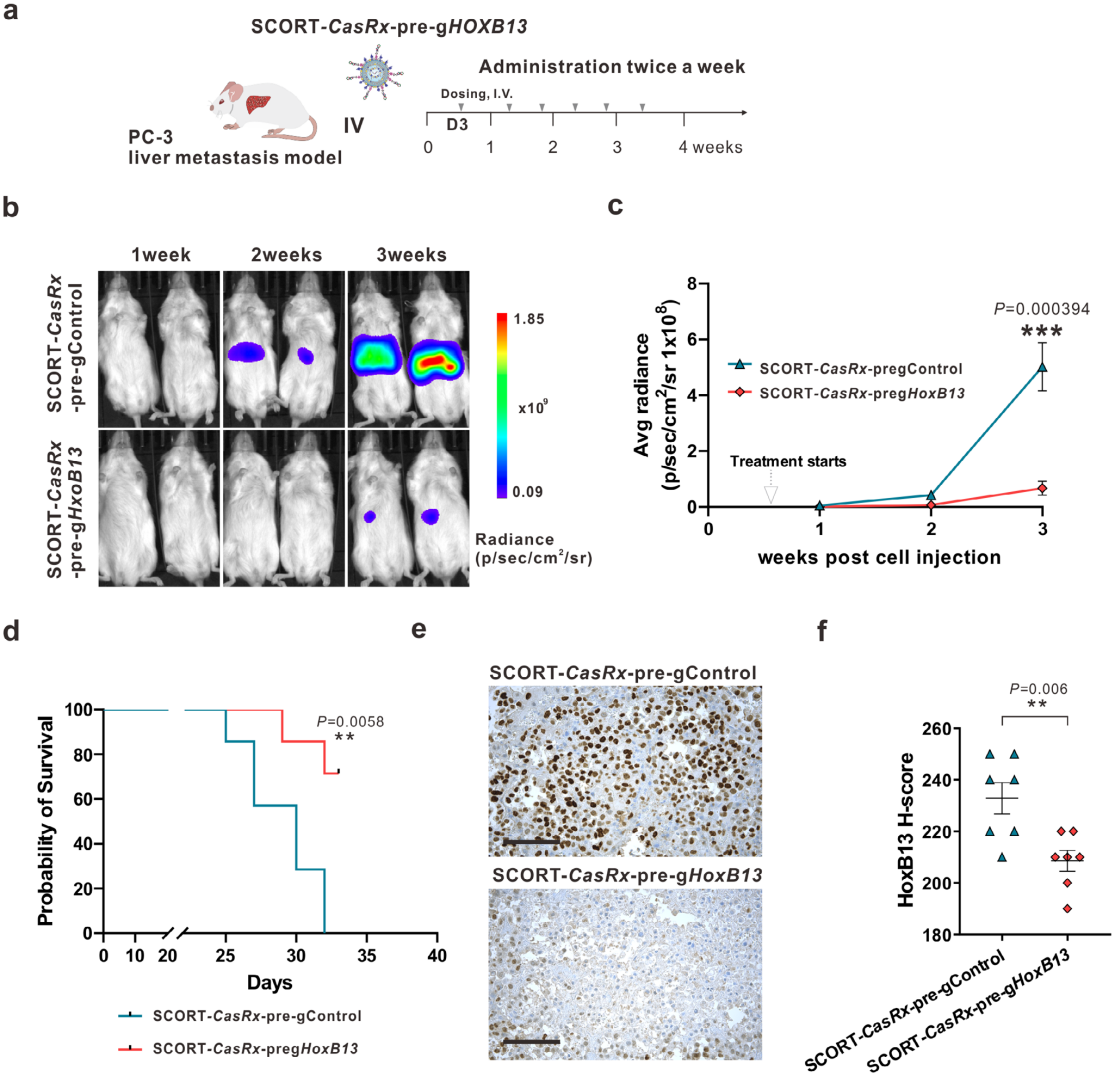
SCORT‐*CasRx*‐pre‐g*HoxB13* treatment inhibits liver metastasis and improves survival in AR‐ liver metastasis mouse model. a) Schematic illustration of the experimental design. The PC‐3 liver metastasis mouse model was established by hemi‐spleen injection of PC‐3 Red‐FLuc cells. Three days after cancer cell engraftment, mice received I.V. administration of SCORT*‐CasRx*‐pre‐gControl, or SCORT*‐CasRx*‐pre‐g*HoxB13*, respectively, twice a week for 3 weeks (*n* = 7). b) Representative bioluminescence imaging of the whole animal on 1, 2, and 3 weeks following cancer cell injection. c) Cumulative luciferase counts during the time course. Data are presented as mean ± SEM. *P* values were calculated by a two‐tailed Student's *t*‐test, ^***^ *p* < 0.001. d) Kaplan‐ Meier survival curves. *P* values were determined by the log‐rank (Mantel‐Cox) test. ^**^ *p* < 0.01. e) Representative images of HoxB13 protein immunostaining. Three days after the final treatment, the liver with metastatic PC‐3 tumors was subjected to IHC analysis. Scale bar: 50 µm. f) H‐score for HoxB13 protein. Data are presented as mean ± SEM. *P* values were calculated by two‐tailed Student's *t*‐test, ^**^ *p* < 0.01.

Next, we established an AR‐ CRPC mouse model via hemi‐spleen injection of PC‐3 cells stably expressing luciferase (PC‐3‐Red‐F‐Luc). Given the lack of variation among the control groups in the AR+ CRPC mouse model, treatments involving SCORT‐*CasRx*‐pre‐gControl and SCORT‐*CasRx*‐pre‐g*HoxB13* were exclusively administered starting three days after cell injection and continued twice per week for three weeks, aligning with the time mice in the control group began to succumb (**Figure** **4**a,d). Similar therapeutic efficacy was observed in this model, as evidenced by bioluminescence measurements at weeks 1, 2, and 3 (Figure 4b,c). Moreover, a significant increase in survival rate was observed following treatment with SCORT‐*CasRx*‐pre‐g*HoxB13* (Figure 4d). Due to the inability to isolate the tumor in this model (i.e., the tumor is diffused in the liver tissue), we restricted our analysis to immunostaining for HoxB13 in the liver sections with metastatic PC‐3 cells. As expected, there was a significant downregulation in HoxB13 expression (Figure 4e,f). Collectively, these findings indicate that SCORT‐*CasRx*‐pre‐g*HoxB13* treatment significantly inhibits both AR+ and AR‐ CRPC liver metastases and extends the mice's survival via downregulation of HoxB13 expression.

### 2.4 Long‐Term SCORT‐*CasRx*‐Pre‐g*HoxB13* Treatment Is Well‐Tolerated In Vivo

**Figure 5:**
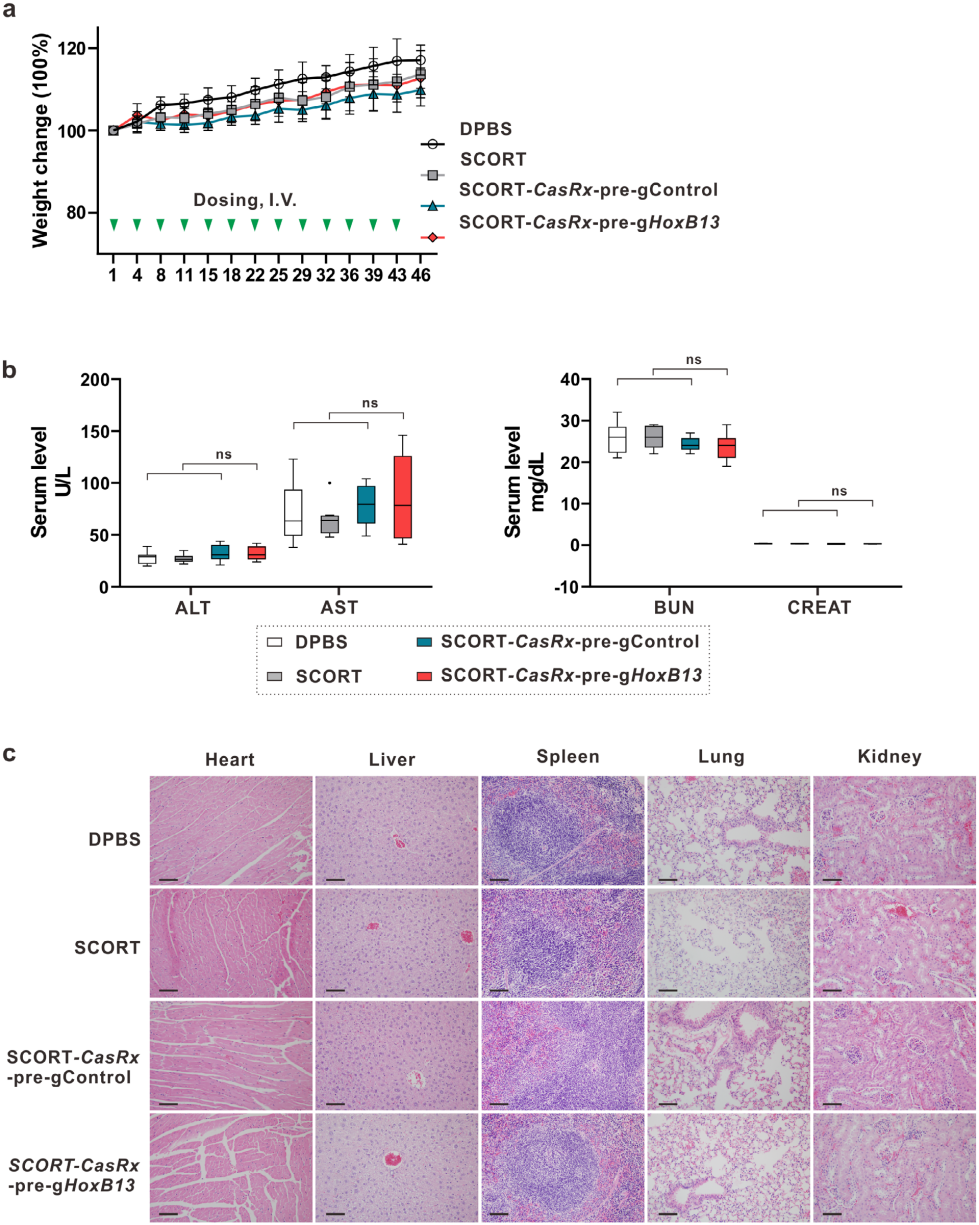
Repeated treatment with SCORT‐*CasRx*‐pre‐g*HoxB13* is well tolerated in immunocompetent mice. CD‐1 mice were given I.V. administrations of DPBS, SCORT LNPs, SCORT*‐CasRx*‐pre‐gControl, and SCORT*‐CasRx*‐pre‐g*HoxB13*, respectively, twice a week for 6.5 weeks (*n* = 8). a) Change in body weight. Body weight at the start of treatment was set at 100%. b) Hepatic and renal functions were not impaired by SCORT‐*CasRx*‐pre‐g*HoxB13* treatment. ALT, alanine transaminase; AST, aspartate aminotransferase; BUN, blood urea nitrogen; CREAT, creatinine. Data are presented as box and whisker plots, where the box represents the median (center line) with bounds of the 25th to 75th percentiles. The whiskers extend to 1.5 times the interquartile range. *P* values were calculated by One‐way ANOVA. ns, not significant. c) No substantial histopathological changes were observed in the indicated organ tissues following SCORT‐*CasRx*‐pre‐g*HoxB13* treatment. Scale bar: 100 µm.

We used normal immunocompetent mice CD‐1 to evaluate the safety of long‐term SCORT‐*CasRx*‐pre‐g*HoxB13* treatment. This study included control groups (DPBS, SCORT LNPs alone, or SCORT‐*CasRx*‐pre‐gControl) and SCORT‐*CasRx*‐pre‐g*HoxB13*, which were administered twice weekly for a period of 6.5 weeks, the maximal duration of treatment used in our studies (Figure 3). SCORT‐*CasRx*‐pre‐g*HoxB13* treatment did not result in evident systemic toxicity (**Figure** **5**). This conclusion is supported by thorough evaluations, including body weight measurements (Figure 5a), liver and kidney function assessments (Figure 5b), histological examinations (Figure 5c), and organ coefficient analyses across major organs such as the heart, liver, spleen, lung, and kidney (Figure S5a, Supporting Information). Furthermore, no notable hematological alterations were observed in the levels of white and red blood cells, platelets, or reticulocytes in mice receiving SCORT‐*CasRx*‐pre‐g*HoxB13*, compared to those in the control groups (Figure S5b, Supporting Information).

Recent studies have indicated that mice and humans can mount innate and adaptive immune responses to Cas9, potentially decreasing therapeutic efficacy and posing significant safety concerns.^[^ ^33^ ^]^ However, it remains unclear whether such immune responses extend to CasRx and other Cas13 enzymes.^[^ ^34^ ^]^ Importantly, we observed no elevation in the levels of any of the 18 chemokines/cytokines examined in plasma in response to long‐term SCORT‐*CasRx*‐pre‐g*HoxB13* treatment (Figure S6, Supporting Information). Conversely, SCORT‐*CasRx*‐pre‐g*HoxB13* treatment led to a reduction in the levels of three factors: KC (keratinocyte‐derived chemokine, also known as IL‐8/CXCL1), macrophage inflammatory protein 2 (MIP‐2, also known as CXCL2), and IL‐12(p70), the heterodimeric form of IL‐12. In addition, no changes were detected in the expression of nine examined chemokines/cytokines in the liver (Figure S7, Supporting Information). These data suggest an absence of an exaggerated immune response or inflammatory reaction in response to SCORT‐*CasRx*‐pre‐g*HoxB13* treatment (Figures S6 and S7, Supporting Information). Altogether, our data demonstrate a robust tolerability of prolonged SCORT‐*CasRx*‐pre‐g*HoxB13* treatment in immune‐competent CD‐1 mice.

### 2.5 Direct Cellular and Transcriptional Outcomes of HoxB13 Knockdown in CRPC Liver Metastases by SCORT‐*CasRx*‐Pre‐g*HoxB13* Treatment

**Figure 6:**
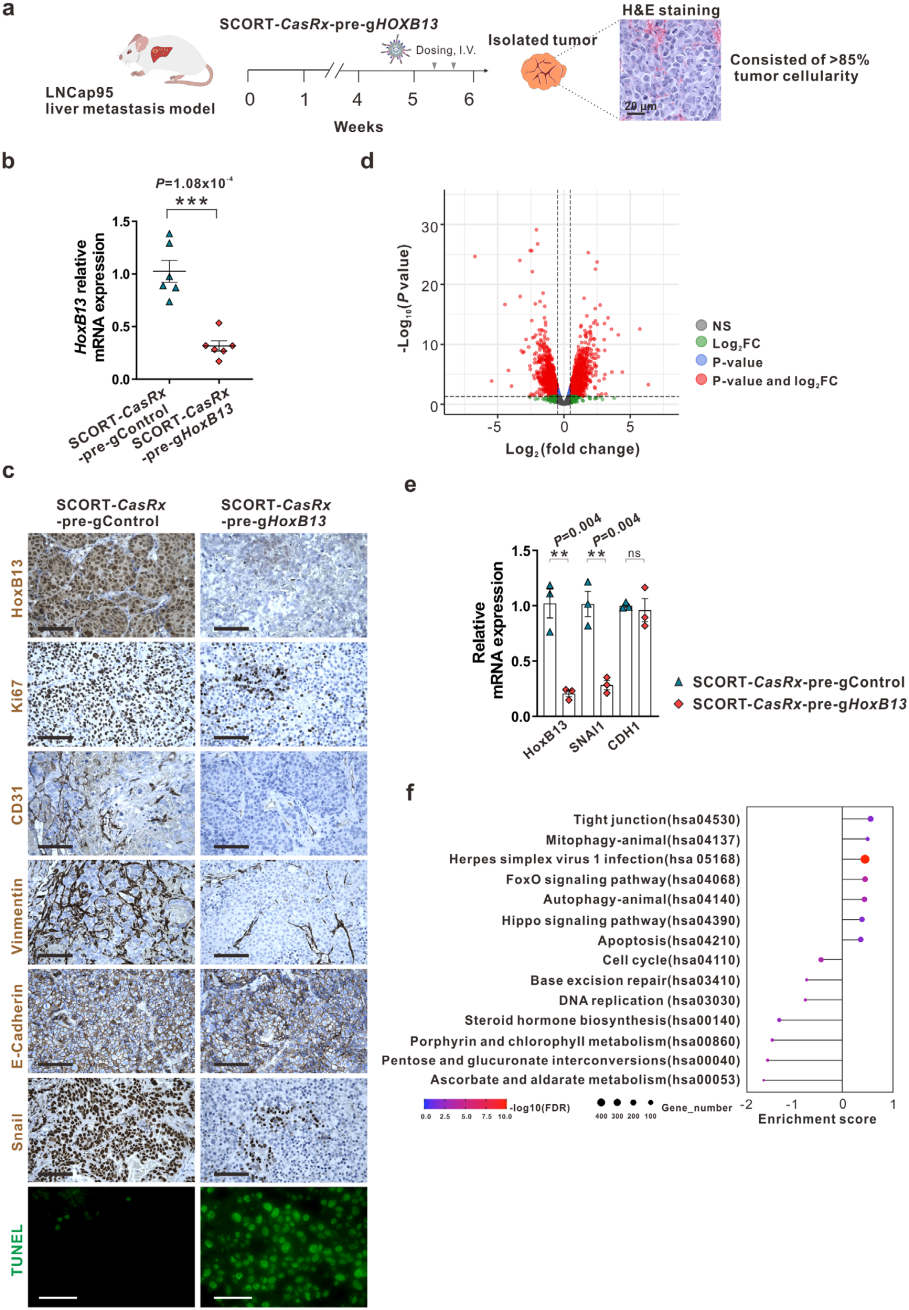
Direct cellular responses and transcriptional changes following SCORT‐*CasRx*‐pre‐g*HoxB13* treatment in AR+ CRPC liver metastasis mouse model. a) Schematic illustration of the experimental design. Five weeks after LNCaP95 cell engraftment, mice received two doses of either SCORT*‐CasRx*‐pre‐gControl or SCORT‐*CasRx*‐pre‐g*HoxB13* with a 3‐day interval. Tumors were isolated three days after the second dose and subjected to analysis (*n* = 6). b) *HoxB13* transcript level. Data are presented as mean ± SEM. *P* values were calculated by One‐way ANOVA, ^***^ *p*<0.001. c) Representative immunostaining images. Scale bar 50 µm. d) Volcano plot of gene expression changes between SCORT*‐CasRx*‐pre‐gControl and SCORT*‐CasRx*‐pre‐g*HoxB13* treated groups. A total of 1,660 upregulated genes and 1,879 downregulated genes were identified and are highlighted in red. e) Validation of *HoxB13*, *SNAI1* (encoding Snail) and *CDH1* (encoding E‐cadherin) gene expression changes in metastatic tumor samples by qRT‐PCR. f) Identified top KEGG pathways by Cistrome GO from DEGs. Pathways associated with positive scores are enriched from the upregulated genes and vice versa. The size of the point represents the number of genes, whereas the color represents the ‐log10 (FDR) value.

We next investigated the mechanisms underlying the organismal phenotypes (i.e., suppression of metastasis and extension of survival time) altered by SCORT‐*CasRx*‐pre‐g*HoxB13* treatment of prostate cancer liver metastases. Recognizing the potential adaptation of cancer cells to long‐term treatment, we opted to study the direct cellular and molecular mechanisms responsible for the therapeutic efficacy of SCORT‐*CasRx*‐pre‐g*HoxB13*. Instead of administering treatment for several weeks, we treated CRPC liver metastasis mouse models with two doses of SCORT‐*CasRx*‐pre‐g*HoxB13* within one week. The mice were sacrificed, and tumors were collected three days following the second dose of treatment (**Figure** **6**a). In isolated tumors from LNCaP95 CRPC liver metastases (6 weeks after hemi‐spleen injection of LNCaP95 cells, Figure 6a), we found that tumors predominantly comprised over 85% LNCaP95 cells and exhibited minimal infiltration of liver tissues, as determined by pathological evaluation (Figure 6a; Figure S8a, Supporting Information). Two‐dose treatments with SCORT‐*CasRx*‐pre‐g*HoxB13* significantly decreased HoxB13 mRNA and protein expression in metastatic LNCaP95 tumors (Figure 6b,c; Figure S8b, Supporting Information). Subsequently, we performed IHC staining of metastatic LNCaP95 tumors using antibodies against various markers associated with proliferation (Ki67), angiogenesis (CD31), and EMT (vimentin, E‐Cadherin, and Snail). Additionally, we carried out a TUNEL assay in conjunction with fluorescence detection to measure apoptosis. The staining for Ki67 decreased, while an increase in green fluorescence was observed in the TUNEL assay in the SCORT‐*CasRx*‐pre‐g*HoxB13* group compared with the SCORT‐*CasRx*‐pre‐gControl group, indicating that SCORT‐*CasRx*‐pre‐g*HoxB13* inhibits cell proliferation and induces apoptosis (Figure 6c; Figure S8b, Supporting Information). Interestingly, vimentin, a canonical marker of EMT, was predominantly expressed in mesenchymal tissues rather than in the metastatic tumor cells themselves, which are primarily surrounded by capillaries within the nests of metastatic cells. Furthermore, the pattern of vimentin expression correlated with the micro‐vessel density (MVD) observed in the corresponding tissues stained for CD31. Notably, SCORT‐*CasRx*‐pre‐g*HoxB13* treatment significantly reduced the expression of extracellular vimentin and CD31, suggesting its inhibition of the angiogenesis associated with metastatic tumors. Intriguingly, while EMT marker Snail expression was significantly decreased following SCORT‐*CasRx*‐pre‐g*HoxB13* treatment, no changes were observed in E‐cadherin expression (Figure 6c; Figure S8b, Supporting Information). This finding, combined with the extracellular expression pattern of vimentin, suggests that SCORT‐*CasRx*‐pre‐g*HoxB13* may suppress the EMT‐independent oncogenic function of Snail rather than EMT.^[^ ^35^ ^]^

We next performed RNA‐seq analysis on metastatic LNCaP95 tumors, revealing 1,660 upregulated genes and 1,879 downregulated genes following SCORT‐*CasRx*‐pre‐g*HoxB13* treatment (Figure 6d; Figure S8c, Supporting Information). Consistent with our finding that SCORT‐*CasRx*‐pre‐g*HoxB13* treatment decreased Snail protein expression (Figure 6c) and the reported role of EMT‐independent oncogenic function of Snail in accelerating cell cycle progression,^[^ ^35^ ^]^ SCORT‐*CasRx*‐pre‐g*HoxB13* treatment reduced the expression of the *SNAI1* gene (encoding Snail protein) and Snail target cell cycle genes (*SKP2*, *MCM3*, and *CCNB1* ^[^ ^35^, ^36^ ^]^). However, it had no effect on the *CDH1* gene (which encodes the E‐Cadherin protein) (Figure 6e; Figure S9a, Supporting Information). In addition, it has no effect (Figure S9a, Supporting Information) on the expression of genes (e.g., *DDX23*, *MEX3B*, and *DHX38*) that show no changes using RNA‐seq‐based off‐target analysis in 293FT cells (Figure 1e; Figure S1a, Supporting Information), further supporting the specificity of CasRx‐mediated HoxB13 targeting. We next curated prostate cancer‐related HoxB13 ChIP‐seq binding peaks from the Cistrome Data Browser^[^ ^37^ ^]^ and integrated these with differentially regulated genes (DEGs) from RNA‐seq to analyze the functions of HoxB13 directly regulated DEGs using Cistrome‐GO‐KEGG pathway analysis.^[^ ^38^ ^]^ This analysis revealed downregulation in cell cycle, base excision repair, and DNA replication pathways, and upregulation in apoptosis, Hippo signaling pathway, and FoxO signaling pathway (Figure 6f), which contributed to the observed decrease in cell growth and an increase in apoptosis.^[^ ^39^ ^]^ Additionally, the downregulation in metabolic processes, including steroid hormone biosynthesis, pentose and glucuronate interconversions, and ascorbate and aldarate metabolism (Figure 6f), can alter energy metabolism, as well as expression and synthesis of factors involved in angiogenesis and extracellular matrix modeling, thereby reducing angiogenesis.^[^ ^40^ ^]^ This decrease is likely indirect, as no direct transcriptional suppression of pro‐angiogenic factors such as VEGF and PDGF were observed (Figure S9b, Supporting Information). Interestingly, among the top five most statistically significantly downregulated genes following SCORT‐*CasRx*‐pre‐g*HoxB13* treatment was the microseminoprotein, prostate‐associated (*MSMP*) gene, which encodes a protein that stimulates angiogenesis through activation of CCR2 signaling.^[^ ^41^ ^]^ Meanwhile, the *UGT2B17* gene, which is highly expressed in CRPC patients,^[^ ^42^ ^]^ has been shown to promote CRPC growth and invasion through activation of the c‐Src kinase.^[^ ^43^ ^]^ RT‐PCR and Western blot analysis of representative HoxB13 target genes from different pathways, using metastatic LNCaP95 tumors, validated our RNA‐seq results (Figures S9a,c‐‐e and S13, Supporting Information). Of note, we discovered that high expression of DNA replication/repair genes, *NEL3* and *LIG1*, which were upregulated by HoxB13, was associated with shorter overall survival of CRPC patients (Figure S10, Supporting Information). Conversely, high expression of the HoxB13‐downregulated gene *ZNF227*, involved in the herpes simplex virus 1 infection pathway, correlated with longer overall survival of CRPC patients (Figure S10, Supporting Information). These associations were consistently observed in analyses of two large CRPC patient cohorts (Figure S10, Supporting Information), highlighting the clinical relevance of genes directly regulated by HoxB13 as identified from metastatic LNCaP95 tumors.

Finally, we investigated the mechanisms contributing to the decreased metastasis and increased survival following SCORT‐*CasRx*‐pre‐g*HoxB13* treatment in the AR‐ CRPC PC‐3 liver metastasis model. Treatments were administered twice within one week, beginning two weeks after cell injection, and liver tissues containing metastatic tumors were collected three days after the final treatment (Figure S11a, Supporting Information). Similar to the findings from the AR+ CRPC LNCaP95 liver metastasis model, proliferation and angiogenesis were suppressed, while apoptosis was induced following treatment with SCORT‐*CasRx*‐pre‐g*HoxB13* (Figure S11b,c, Supporting Information). Different from LNCaP95 metastatic tumor cells, vimentin was primarily expressed in the cytosol of metastatic PC‐3 tumor cells, and its expression decreased following treatment with SCORT‐*CasRx*‐pre‐g*HoxB13*, suggesting a decrease in tumor growth and metastasis. Remarkably, Snail was strongly expressed in control tumors, and its expression dramatically decreased following treatment, whereas E‐Cadherin was very weakly expressed, and its expression decreased rather than increased following treatment (Figure S11b,c, Supporting Information), suggesting that SCORT‐*CasRx*‐pre‐g*HoxB13* treatment also inhibits the non‐canonical oncogenic function of Snail^[^ ^35^ ^]^ in PC‐3 tumors.

Collectively, our findings demonstrate that SCORT‐*CasRx*‐pre‐g*HoxB13* treatment decreases metastatic tumor growth and angiogenesis while promoting apoptosis of both AR+ and AR‐ CRPC.

## 3 Discussion

Effective therapeutics for metastatic cancer require approaches that can simultaneously target diverse cancer hallmarks, such as uncontrolled cell growth, resistance to cell death, induced angiogenesis, activated metastasis, and deregulated cellular metabolism.^[^ ^44^ ^]^ TFs are ideal therapeutic targets for metastatic cancers because they are master regulators, controlling hundreds to thousands of downstream target genes involved in multiple signaling pathways contributing to cancer hallmarks.^[^ ^4^, ^5^, ^7^ ^]^ HoxB13, a prostate‐specific lineage TF,^[^ ^45^ ^]^ is highly expressed in CRPC and promotes CRPC growth, invasion, and metastasis^[^ ^20^ ^]^ (Figure 1). Unfortunately, like the majority of TFs, HoxB13 is considered untargetable by traditional small molecule‐based drug design. To overcome this challenge, we chose to use the RNA‐targeting CRISPR/CasRx system, previously employed for programmable RNA knockdown in both in vitro and in vivo studies by us and others, demonstrating minimal off‐target effects.^[^ ^11^, ^13^ ^]^ Consistently, we found that CasRx, guided by a pre‐gRNA targeting *HoxB13* mRNA, mediates a potent and specific knockdown of HoxB13 in vitro (Figure 1; Figure S1, Supporting Information).

To effectively target undruggable TFs like HoxB13 in metastatic cancer cells in vivo, precise delivery of CasRx and pre‐gRNAs to tumor cells within organs is essential. Although the small size (2.9 kb) of the Cas13d enzyme facilitates its packaging into adeno‐associated virus (AAV) vectors,^[^ ^11^ ^]^ AAV‐based therapies often face limitations including immunogenicity (especially upon repeated injections), a small fraction of tumor cells transduced, production difficulties, and potential tumorigenicity.^[^ ^46^ ^]^ By contrast, conventional LNPs offer a non‐viral platform but typically rely on passive biodistribution, leading to nonspecific uptake—primarily in the liver—rather than active targeting of metastatic tumors.^[^ ^47^ ^]^ To overcome these challenges, we developed the SCORT LNP system to preferentially deliver *CasRx* mRNA/pre‐gRNA to metastatic CRPC cells in the liver rather than to surrounding liver cells or other organs (Figure 2; Figure S3d, Supporting Information). This improved targeting arises from three key design elements: (1) our ionizable lipid FTT5^[^ ^15b^ ^]^ for potent in vivo RNA delivery, (2) a low molar ratio of PEG to enhance CRPC cell uptake in both culture and liver environment,^[^ ^27^, ^48^ ^]^ and (3) an E3 aptamer on the LNP surface binds to TfR1 that highly expressed by CPPC cells^[^ ^25^ ^]^ (Figure 2). Notably, the E3 aptamer also recognizes other TfR1‐positive cancer cells that frequently metastasize to the liver, such as colorectal, pancreatic, lung, and breast cancer,^[^ ^25^, ^26^ ^]^ suggesting the broader applicability for SCORT in targeting metastatic cancers. Meanwhile, although SCORT LNPs may require repeated dosing for sustained therapeutic impact, AAV vectors can achieve longer‐lasting expression from a single dose,^[^ ^49^ ^]^ albeit with the aforementioned drawbacks. Additionally, the SCORT platform allows flexibility in organ targeting by customizing lipid composition (e.g., incorporating a 50% molar ratio of DOTAP, a selective organ targeting (SORT) molecule^[^ ^50^ ^]^) to reach extrahepatic metastatic sites such as the lungs.^[^ ^51^ ^]^ Ongoing optimization of SCORT components could further refine selectivity and efficacy across different tumor models.

**Figure 7:**
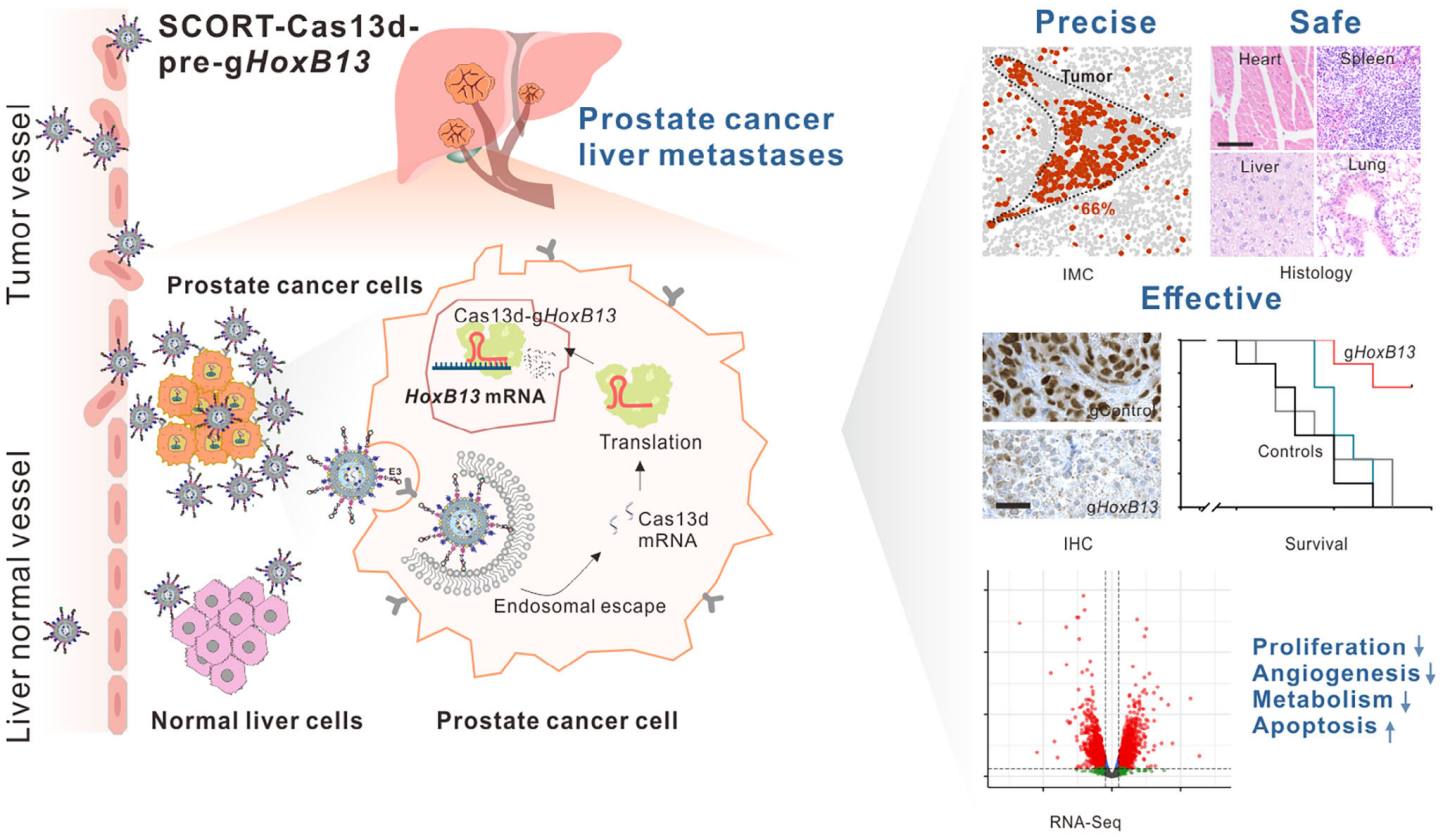
Schematic overview of the SCORT–*Cas13d*–a pre‐g*HoxB13* approach for targeting metastatic prostate cancer in the liver. Intravenously administered SCORT nanoparticles carry *Cas13d* mRNA and pre‐guide RNAs (pre‐g*HoxB13*), which preferentially accumulate in HoxB13‐dependent prostate cancer metastases rather than in normal liver cells. Within metastatic tumor cells, the *Cas13d* mRNA is translated, and pre‐g*HoxB13* is processed to generate mature g*HoxB13* guides. The resulting Cas13d–g*HoxB13* complexes knock down *HoxB13* transcripts, leading to reduced HoxB13 protein expression. Notably, the systemic treatment showed no significant adverse effects on major organs (e.g., heart, liver, spleen, and lung) and did not raise immune markers, underscoring the approach's safety. Immunohistochemistry (IHC) confirmed efficient HoxB13 knockdown in vivo, and survival analyses demonstrated improved outcomes following repeated SCORT–*Cas13d*–pre‐g*HoxB13* administration. Transcriptomic profiling further revealed that SCORT–*Cas13d*–pre‐g*HoxB13* modulates multiple gene pathways (Figure 6), reducing tumor proliferation, angiogenesis, and metabolic activity while promoting apoptosis. Overall, these findings suggest that SCORT–*Cas13d*–pre‐g*HoxB13* is a precise, safe, and effective method for silencing the “undruggable” transcription factor HoxB13 in metastatic prostate cancer.

Importantly, systemic treatment with SCORT‐*CasRx*‐pre‐g*HoxB13* effectively suppresses CRPC metastases and extends the survival in both AR+ and AR‐ CRPC mouse models with liver metastasis (Figures 3 and 4). This outcome results from decreased tumor growth and angiogenesis, and increased apoptosis (Figures 6 and 7), underscoring the therapeutic importance of targeting master TFs contributing to various cancer hallmarks. Consistent with its role in inhibiting multiple tumor cellular phenotypes, SCORT‐*CasRx*‐pre‐g*HoxB13* treatment downregulates several oncogenic pathways and upregulates multiple tumor suppressive gene pathways in AR+ CRPC tumors (Figure 6). Due to the significant infiltration of liver tissues into the AR‐ CRPC tumors (Figure S11a, Supporting Information), isolating AR‐ tumors with a high percentage of tumor cells, as in AR+ CRPC tumors, for bulk RNA‐seq was challenging. Future studies using single‐cell RNA‐seq will aim to overcome this limitation. In addition, considering CRPC patients who have liver metastases often also harbor bone and other soft tissue metastases, this illustrates the need for future combination approaches with our liver‐directed therapy or broader selective targeting of more metastatic sites. Interestingly, although Snail and E‐cadherin are well‐known EMT markers—with Snail known to transcriptionally repress E‐cadherin expression^[^ ^52^ ^]^—we discovered that SCORT‐*CasRx*‐pre‐g*HoxB13* treatment significantly decreases Snail expression without enhancing E‐cadherin expression (Figure 6; Figure S8,11, Supporting Information). Moreover, this treatment reduces the expression of the S*NAI1* gene itself and directly targets cell cycle genes such as *SKP2, MCM3*, and *CCNB1* (Figure 6; Figure S9a, Supporting Information). Together, these results suggest that SCORT‐*CasRx*‐pre‐g*HoxB13* treatment inhibits the EMT‐independent, non‐canonical oncogenic function of Snail.^[^ ^35^ ^]^ Since there are no HoxB13 binding sites close to the S*NAI1* gene,^[^ ^20a^ ^]^ it is plausible that HoxB13 indirectly regulates the *SNAI1* gene by directly regulating other factors. Future studies will explore these mechanisms.

Repeated dosing is typical in cancer treatment and for RNA‐based therapies, emphasizing the paramount importance of safety considerations. To the best of our knowledge, there has been no systematic safety evaluation of Cas13 mRNA‐based repeated therapies in mice. Importantly, our repeated SCORT‐*CasRx*‐pre‐g*HoxB13* treatment (administered twice per week for 6.5 weeks) was well‐tolerated in normal CD‐1 mice, as evidenced by no significant changes in body weight, liver and kidney functions, or histology of major organs including the heart, liver, spleen, lung, and kidney. Additionally, there was no elevation in the levels of examined chemokines/cytokines in response to prolonged SCORT‐*CasRx*‐pre‐g*HoxB13* treatment (Figure 5; Figure S5,S6,S7, Supporting Information). We reason that the lack of toxicity from prolonged SCORT‐*CasRx*‐pre‐g*HoxB13* treatment can be attributed to the following reasons: 1) the nanomaterials for constructing SCORT LNP are safe, as repeated injections of empty SCORT LNP exhibit no toxicity (Figure 5; Figure S5,S6,S7 Supporting Information); 2) the high specificity of CasRx in mammalian cells^[^ ^11^, ^13^ ^]^ (Figure 1e, Figure S1a, Supporting Information) minimizes potential toxicity associated with promiscuous RNA cleavage by some other Cas13 enzymes, such as Cas13a in cancer cells^[^ ^53^ ^]^; 3) unlike Cas9 protein derived from the bacterial pathogens *Staphylococcus aureus* and *Streptococcus pyogenes*, which may cause preexisting immunity,^[^ ^33a,b^ ^]^ CasRx is derived from *Ruminococcus flavefaciens*, a primary degrader of plant structural carbohydrates in the rumen of mammals and an apparently non‐pathogenic bacterium.^[^ ^54^ ^]^ Indeed, we found that repeated SCORT‐*CasRx*‐pre‐g*HoxB13* treatment does not stimulate the expression of inflammatory cytokines/chemokines (Figures S6 and S7, Supporting Information); 4) HoxB13 is only strongly expressed in prostate tissues.^[^ ^45^ ^]^

In summary, we have overcome the challenge of directly targeting the undruggable TF HoxB13 in metastatic cancer by developing the SCORT‐*Cas13d‐gHoxB13* system (**Figure** **7**). This broadly applicable technology holds great promise for both translational and fundamental research by enabling the targeting and investigation of other previously undruggable oncogenic TFs in metastatic cancers, through the design of new gRNAs and the modification of nanomaterials.

## 4 Experimental Sections

### Human Pathology

Paraffin‐embedded tissue microarray sections with multiple cores of prostate tumors (Normal, ADPC, mCRPC) were constructed by Dr. Jiaoti Huang (Duke). The tissues were obtained from Duke University's tissue bank (known as the Biorepository and Precision Pathology Center [BRPC]) and are labeled with study IDs only (e.g., sample 001, 002, etc.). The researchers have no access to patient identifiers (e.g., name, date of birth). A detailed data security plan has been implemented. Data files are stored on a password‐protected computer located in the locked, private office of Dr. Jiaoti Huang, Chairman of the Department of Pathology at Duke University School of Medicine. Duke provides security patrols, and public areas are constantly monitored. The level of HoxB13 expression was assessed via immunohistochemical staining, wherein HoxB13 immunoreactivity was examined and scored. The expression score was derived by combining both the intensity and the percentage of immunoreactive cells.

### LNP Preparation and Characterization—LNP Preparation and Conjugation

The preparation procedure for LNP candidates was followed as previously described.^[^ ^13b^ ^]^ Briefly, a lipid‐like compound functionalized TT derivative 5 (FTT5, synthesized by Dr. Yizhou Dong's laboratory at Icahn School of Medicine at Mount Sinai),^[^ ^15b^ ^]^ DOPE (Avanti Polar Lipids, no. 850725), cholesterol (Sigma, no. C3045), DMG‐PEG2000 (NOF America Corporation), and DSPE‐PEG‐maleimide (Avanti Polar Lipids, no.880126) were dissolved in ethanol in molar ratios of 20:30:39:0.75:1, 20:30:38.25:1.5:1, 20:30:36.75:3:1, respectively. RNAs were diluted in citrate buffer (10 mM, pH 4.0). The prepared solutions were mixed via a microfluidic mixing device (The NanoAssemblr Spark^TM^, Precision Nanosystems) at a volume ratio of 1:2 (organic: aqueous) to achieve a final weight ratio of lipids: RNAs of 20:1. Luciferase mRNA (no. L‐7202), mCherry mRNA (no. L‐7203), EGFP mRNA (no. L‐7601) were purchased from TriLink. CasRx mRNA with pseudouridine modification (pseudouridine‐5′‐triphosphate, TriLink) was synthesized using in vitro transcription (AmpliScribe T7‐Flash Transcription Kit, Lucigen) and was then installed with 5′ cap (Vaccinia Capping System, NEB; Cap 2′‐O‐methyltransferase, NEB) and 3′ poly(A) tail structures by Dr. Yizhou Dong's laboratory.^[^ ^55^ ^]^ Pre‐gControl and pre‐*gHoxB13* oligos with 2’OMe and phosphorothioate modification were synthesized by TriLink, and their sequences are listed in Extended Data Table S1 (Supporting Information). The buffer was exchanged to DPBS containing 5 mM EDTA by 7K Zeba Spin Desalting Columns (ThermoFisher Scientific, no. 89882), incubated with a fivefold molar excess of E3 aptamer compared to DSPE‐PEG‐maleimide for 2 h at room temperature followed by overnight at 4 °C with gentle shaking for the thiol‐maleimide cross‐linking reaction. The E3 aptamer, with 2’ Fluoro and 5’ C6 thiol linker modification, was synthesized by TriLink; the sequence is included in Extended Data Table S1 (Supporting Information). The free aptamer was separated and washed by Amicon Ultra spin columns (10k, no. UFC901024) the following day. The RNA dose for the animal studies (*CasRx* mRNA: pre‐gRNA, 1:2, wt: wt) is 1.5 mg kg^−1^.

### LNP Characterization

The Zetasizer Nano ZS (Malvern Panalytical) was used to measure the particle size, polydispersity index (PDI), and zeta potential. Transmission Electron Microscopy (TEM) examination was performed by Chapel Hill Analytical and Nanofabrication Laboratory at the University of North Carolina‐Chapel Hill, using a Talos F200X (Thermo Fisher) at an accelerating voltage of 200 kv. mRNA encapsulation efficiency was determined by the RiboGreen Assay. Briefly, mRNA concentration was measured using the Quant‐iT RiboGreen RNA Assay Kit (Thermo Fisher, no. R11490) following the manufacturer's instructions. Briefly, mRNA‐encapsulated LNPs, either treated with diluted Triton X‐100 or without Triton treatment, were incubated with the RiboGreen reagent for 2–5 min at room temperature, protected from light. Fluorescence was measured using a SpectraMax M3 microplate reader (Molecular Devices) with excitation at ≈480 nm and emission at ≈520 nm. mRNA concentrations were determined using a standard curve generated from known RNA standards.

### In Vitro Evaluation of Cell Delivery, Proliferation, and Invasion—Cell Lines and Reagents

293FT cells were obtained from Invitrogen (no. R70007), and PC‐3 cells were obtained from the American Type Culture Collection (ATCC, no. CRL‐1435). Both cell lines were grown in Dulbecco's modified Eagle's medium (DMEM) supplemented with 10% fetal bovine serum (FBS, Gibco, no. A4766801). LNCaP95 cells, provided by Dr. Jun Luo (Johns Hopkins University), were grown in phenol red‐free RPMI 1640 medium with 10% charcoal‐stripped FBS. LNCaP95 cells stably expressing luciferase (LNCaP95‐Luc) were generated by incubating LNCaP95 cells with IVISbrite Red F‐luc‐Puromycin Lentiviral Particles (no. CLS96002), followed by selection with puromycin (Gibco, no. A1113803,1ug mL^−1^). PC3 Red‐Fluc was obtained from PerkinElmer (no. BW128444) and was grown in Eagle's Minimum Essential Medium (EMEM) supplemented with 10% FBS. AML12 cells and THLE2 cells were obtained from Duke University Cell Culture Facility, and sourced from ATCC. AML12 cells were grown in DMEM/F12 medium with 10% FBS, 1x Insulin‐Transferrin‐Selenium (ITS), and 40 ng mL^−1^ Dexamethasone. THLE2 cells were cultured in BEGM (Lonza, no. CC3170, the BEGM kit from which gentamycin/amphotericin and epinephrine were discarded) supplemented with 10% FBS, 5 ng mL^−1^ EGF and 70 ng mL^−1^ phosphoethanolamine. All cell lines were maintained at 37 °C with 5% CO_2_. The cell lines were routinely tested for mycoplasma contamination using the VenorTM GeM Mycoplasma Detection Kit (Sigma‐Aldrich, no. MP0025) following the manufacturer's protocol.

### Cell Delivery Evaluation

LNCaP95 cells (3 × 10^5^), PC‐3 cells (2 × 10^5^), or AML12 cells (2 × 10^5^) were seeded into 6‐well plates and allowed to reach 70–80% confluence overnight, followed by incubation with SCORT LNP candidates encapsulating 1 µg luciferase mRNA for 24 h. For subsequent analysis, LNCaP‐95 cells (1 × 10^4^), PC‐3 cells (6 × 10^4^), or AML12 cells (3 × 10^4^) were reseeded into 96‐well plates with black walls and clear bottoms (Corning, no. 3603). They were then incubated with 150 ng µL^−1^ IVIS brite D‐Luciferin Potassium Salt Bioluminescent Substrate (Perkin Elmer, no. 122799) for 5 min. The luminescence signal was then measured using the Spectra Max M3 microplate reader and imaged using the IVIS lumina XR system (Caliper Life Science). For the flow cytometry assay, the three cell lines were prepared in 6‐well plates overnight until 70–80% confluence. They were then separately incubated with SCORT LNP candidates encapsulating 1 µg of EGFP mRNA (TriLink, no. L‐7601) or mCherry mRNA (TriLink, no. L‐7203) for 24 h. Subsequently, fluorescence analysis was conducted using the Fortessa x20 Analyzer (BD).

### Cell Proliferation and Invasion Assays

LNCaP95 cells (3 × 10^5^) or PC‐3 cells (2 × 10^5^) or AML12 cells (2 × 10^5^), or THLE2 cells (2 × 10^5^) were seeded into 6‐well plates and allowed overnight to reach 70–80% confluence. The cells were then transfected with 1 µg /mL^−1^ *CasRx* mRNA along with 2 µg mL^−1^ of either pre‐gControl oligo or pre‐g*HoxB13* oligo using Lipofectamine MessengerMAX (Invitrogen, no. LMRNA015). Opti‐MEM (Mock) and Lipofectamine MessengerMAX reagent alone (eLNPs) served as controls. Cell proliferation was assessed using the WST‐1 assay. The following day, LNCaP95 cells (5 × 10^3^) and PC‐3 cells (3 × 10^3^) were reseeded into 96‐well plates. On day 1, day 3, and day 5 post‐transfection, cell proliferation reagent WST‐1 (10 µL per well, Roche, no. 11644807001) was added, and absorbance at 450 nm was measured using the Spectra Max M3 microplate reader after a 1‐h incubation. Cell invasion was evaluated using a transwell assay. After 48 h of treatments, LNCaP95 cells (3 × 10^5^) and PC‐3 cells (2 × 10^5^) were suspended in FBS‐free medium and seeded into 8‐µm Transwell inserts (Corning, no. 3422) coated with Matrigel (Corning, no. 356237). After 24 h, the invasive cells were fixed with methanol and stained with crystal violet (Sigma, no. 1159400025) for 20 min. Cells on the upper surface of the membranes were removed with a cotton swab, while those that had penetrated were photographed and counted.

### In Vivo Studies: CRPC Mouse Models For Therapeutic Evaluation and CD‐1 Mice for Toxicity Profiling

For all animal studies in this research, the study protocols were approved by the Institutional Animal Care and Use Committee of Duke University under protocol number A176‐21‐08‐24.

### Mice

CD‐1 mice (strain no. 022, males, aged 5–8 weeks) were purchased from Charles River (Wilmington, MA). NSG (NOD.Cg‐Prkdc^scid^ Il2rg^tm1Wjl^/SzJ, strain no. 005557, male, 4–6 weeks old) were acquired from The Jackson Laboratory (Bar Harbor, ME) or Rodent Gnotobiotic and Breeding Core at Duke University Medical Center. The mice were maintained under pathogen‐free conditions (22.5 °C, 51% humidity, and a 12h light/dark cycle) and were acclimatized for 1 week before the start of the studies.

### Castration and Liver Metastatic CRPC Models

NSG Mice were surgically castrated as previously described.^[^ ^56^ ^]^ After 1–2 weeks of recovery, a liver metastatic CRPC model was established by hemispleen injection of LNCaP95‐Luc Cells (1 × 10^6^ per mouse) or PC‐3 Red‐Fluc cells (3 × 10^5^ per mouse), following previously described methods.^[^ ^32^ ^]^

### Treatment Regimens—*for Metastasis and Survival Studies*

In the LNCaP95 mouse model, treatments including DPBS, SCORT LNP, SCORT‐*CasRx*‐pre‐gControl, and SCORT‐*CasRx*‐pre‐g*HoxB13* were respectively administered to mice (*n* = 7 per group) starting one week after cell injection, with a frequency of twice per week for 6.5 weeks. Bioluminescent signal measurements were performed at 3‐, 5‐, and 7‐weeks post‐cell injection to monitor metastasis. For the PC‐3 mouse model, treatments of SCORT‐*CasRx*‐pre‐gControl and SCORT‐*CasRx*‐pre‐g*HoxB13* commenced 3 days after cell injection, administered twice per week for 3 weeks. Bioluminescent signal measurements were taken at 1‐, 2‐, and 3‐weeks post‐cell engraftment. For both models, animals were monitored daily until death or humane euthanasia. Tumor tissues or liver tissues with metastatic tumors were collected 72 h after the last treatment for histological and/or biochemical analysis.

### Treatment Regimens—*for Mechanistic Studies*

In the LNCaP95 mouse model (5 weeks after cell injection), mice received treatments of SCORT‐*CasRx*‐pre‐gControl and SCORT‐*CasRx*‐pre‐g*HoxB13* twice within one week, with a 3‐day interval between doses. Tumor tissues were collected 72 h after the final treatment for subsequent analysis, including molecular analysis, biochemical assessments, and histological examinations. Similarly, in the PC‐3 mouse model (2 weeks after cell injection), mice underwent the same treatment regimen as the LNCaP95 mouse model. Liver tissues with metastatic tumors were collected 72 h after the final treatment for histological analysis.

### IVIS Imaging

For monitoring the luciferase signal, LNCaP95 or PC‐3 mouse models were intraperitoneally injected with substrate D‐Luciferin (150 mg kg^−1^, Pekin Elmer, no. 770505) for bioluminescence detection in the whole body or specific organs. For mCherry signal visualization, organs in the LNCaP95 mouse model were imaged for fluorescence 2 h after SCORT‐mCherry injection. All signal measurements were conducted using the IVIS Lumina XR system (Caliper Life Science).

### Imaging Mass Cytometry (IMC)

The LNCaP95 tumor tissues were processed and stained per protocol as previously described.^[^ ^57^ ^]^ Briefly, tumor sections were baked at 60 °C overnight, then dewaxed in xylene and rehydrated in a graded series of alcohol. Heat‐induced epitope retrieval was performed in an EZ‐Retriever System (BioGenex) at 95 °C in Citrate buffer at pH 6 for 20 min. After immediate cooling for 20 min, the sections were blocked with 3% BSA in TBS for 1 h and then incubated overnight at 4 °C with an antibody master mix. The Samples were subsequently washed four times with TBS/0.1% Tween20 before staining with Cell‐ID Intercalator (Standard BioTools) for 5 min for nuclear staining. Slides were washed twice with TBS/0.1% Tween20 and air dried to store at 4 °C for ablation. The sections were ablated with Hyperion Imaging System (Standard BioTools) for data acquisition. The raw data are preprocessed and checked for tissue integrity, staining quality, and signal range prior to downstream analysis. For every Region of interest (ROI), the single cells are segmented using *ilastik* ^[^ ^58^ ^]^ and *CellProfiler*,^[^ ^59^ ^]^ based on DNA staining (Ir191) and cell surface markers (i.e., HNF4a, PSMA, and F4/80). Each ROI contains an average of 2,093 cells within the area of 0.25 mm^2^. Following cell segmentation, data are processed and visualized using the Histology Topography Cytometry Analysis Toolbox (HistoCAT).^[^ ^60^ ^]^ The mean intensities of each marker for all single cells are extracted and consolidated in R scripts for downstream analysis. The positive cells for each marker were determined by thresholding method based on histogram and cross‐validated by unsupervised clustering from normalized intensity values. Cell densities or percentages of each cell type are calculated by normalizing cell counts by ROI areas or total cell counts of that ROI.

### Toxicity Profiling

Male CD‐1 mice (*n* = 8 per group) were administered DPBS, SCORT LNP, SCORT‐*CasRx*‐pre‐gControl, and SCORT ‐*CasRx*‐pre‐g*HoxB13*, respectively, twice per week for 6.5 weeks. Body weight was measured every 3–4 days. Blood samples were collected 72 h after the last treatments. Complete blood cell counts (CBC) were assessed using IDEXX Procyte DX (IDEXX Laboratories). Serum levels of alanine aminotransferase (ALT), aspartate aminotransferase (AST), blood urea nitrogen (BUN), and creatinine (CREAT) were measured by the Animal Histopathology and Laboratory Medicine Core at the University of North Carolina‐Chapel Hill. Additionally, plasma levels of 18 cytokines/chemokines were analyzed by Alfawasserman Vet Axcel (Alfawasserman Diagnostic Technologies) using the MAGNETIC Kit (Millipore Sigma, no. MCYTOMAG‐70K). Major organ tissues, including the lungs, liver, spleen, heart, and kidneys, were collected for organ coefficient evaluation and histopathological examination.

### RNA Isolation and Quantitative RT‐PCR (qRT‐PCR)

Total RNA of treated cells or tumor tissues was isolated using the RNeasy Mini Kit (Qiagen, no.74106) and was reverse transcribed to cDNA using the High‐Capacity cDNA Reverse Transcription Kit (Life Technologies, no. 4368814). qRT‐PCR was performed with PowerUp SYBR Green PCR Master Mix reagents (Applied Biosystems, no. A25742) on a qTOWER^3^G system (Analytik Jena). The primers used were synthesized by IDT, and their sequences are listed in Extended Data Table 2. The transcript level was normalized by the internal control, *18s‐rRNA*.

### RNA‐Sequencing (RNA‐seq) and Data Analysis

RNA‐seq analysis was conducted following previously established procedures.^[^ ^20a^ ^]^ Briefly, 293FT cells were transfected with either a pre‐gControl or a pre‐HoxB13 vector in combination with a CasRx expression plasmid for 24 h. LNCaP95 liver metastasis samples were collected for mechanistic investigation assays. RNA extraction was performed using the RNeasy Mini Kit (Qiagen, no. 74106). To generate libraries, mRNA enrichment was carried out using the NEBNext Poly(A) mRNA Magnetic Isolation Module (NEB, no. E7490L). After two rounds of processing, the enriched mRNA was subject to library construction with NEBNext Ultra II Directional RNA Library Prep Kit for Illumina (NEB, no. E7765S) according to the manufacturer's instructions. Complementary DNA molecules were amplified for eight cycles by PCR. The resulting non‐size‐selected libraries were sequenced on either an Illumina NovaSeq 6000 or NovaSeq X Plus platform at the Duke Sequencing and Genomic Technologies Shared Resource. For the RNA‐seq data analysis of HoxB13 knockdown in 293FT cells, alignment of reads to the human hg19 reference genome was executed using HISAT2 v.2.1.0, and expression counts were calculated using htseq‐count v.0.11.2. Differentially expressed genes (DEGs) were identified using DESeq2 v.1.26.0^[^ ^61^ ^]^ with a cutoff of absolute fold change > 2 and a *q* value < 0.01. For the RNA‐seq analysis of isolated tumors from LNCaP95 CRPC liver metastases, the fastq files were first performed quality control by Trim Galore v.0.6.10^[^ ^62^ ^]^ and were then mapped to hg38 by Hisat2 v.2.2.1^[^ ^63^ ^]^ with its default parameters. The read counts for each gene were calculated by featureCounts v.2.0.6^[^ ^64^ ^]^ with parameters ‐s 2 and ‐M. DEGs were identified by DESeq2 v.1.40.1^[^ ^61^ ^]^ with the cutoffs of absolute fold change ≥ 1.4 and *p*‐value ≤ 0.05.

### Cistrome‐GO Analysis

All HoxB13 peaks associated with prostate cancer were curated, including those from cell lines and tissues (CistromID: 56663, 56664, 84204, 84205, 84342, 84343, 84346, 84347, 88278, 88495, 88496, 88497, 88686, 89854, 89856, 89857, 90052, 90057, 90058, 93209), using Cistrome Data Browser v2.0.^[^ ^37^ ^]^ The separated peak files were further merged into a single file using bedtools^[^ ^65^ ^]^ merge with default parameters. Subsequently, Cistrome GO analysis was performed on the Cistrome GO web server^[^ ^38^ ^]^ by inputting both HoxB13 peaks and DEGs from RNA‐seq with the following advance setting: Peak number to use 100 000; customized half‐decay distance: 50; cutoffs of (logFoldChange, FDR) for DEGs: 0.4/0.05; FDR cutoff of GO/KEGG terms to return: 0.2; Minimum and maximum gene number in GO and KEGG gene sets: 10/2000. For visualization convenience, the enrichment scores calculated in Cistrome GO were further scaled by log10.

### Western Blotting (WB)

The treated Cells or homogenized tumor tissues were collected and lysed in RIPA buffer (Boston BioProducts, no. BP115) with 1X cOmplete protease inhibitor cocktail (Roche, no.11697498001) for 30 min on ice. The protein concentration was determined by a BCA protein assay (Thermo, no.23225). Samples were resolved on 4–15% Mini‐PROTEAN TGX Stain‐Free Protein Gels (Bio‐Rad, no. 4568084) or 4–15% Criterion TGX Precast Midi Protein Gel (Bio‐Rad, no.5671085), then transferred onto PVDF membranes (Bio‐Rad, no.1704157, no. 88520). After being blocked with 5% milk powder solution (Bio‐Rad, no. 170–6404) for 1 h, the membranes were probed with HoxB13 antibody (Santa Cruz, no. SC66923, 1:200; or GeneTex, no. GEX129245, 1:500), MSMP antibody (Thermo Fisher, no. PA5‐63079, 1:250) UGT2B17 (Thermo Fisher, no. PA5‐13430, 1:1000), PRKACB (Thermo Fisher, no. 12232‐1‐AP, 1:500) or calnexin antibody (Enzo Life Sciences, no. ADI‐SPA‐860‐F, 1:1000) overnight at 4 °C. Following incubation with corresponding secondary antibodies (LI‐COR, no. 926–80011, 1:5000), immunoblots were developed using Supersignal West Pico PLUS chemiluminescent substrate and visualized using the C‐DiGit Chemiluminescent Western Blot Scanner (Li‐COR). Protein expression was quantified by densitometry (Image J 1.52a/Java 1.8.0_112) and normalized to calnexin.

### Immunohistochemistry (IHC) and Scoring

LNCaP95 tumor tissues or PC‐3 metastatic liver were fixed in 4% paraformaldehyde, routinely processed in paraffin, and sectioned at 4 µm for immunostaining. Briefly, sections were deparaffinized, and antigen retrieval was performed using citrate buffer (Abcam, no. AB93678), followed by blocking with 1.2% H2O2. The sections were then incubated overnight at 4 °C with the following primary antibodies: anti‐HoxB13 (Santa Cruz, no. SC66923, 1:200), anti‐CD31 (Abcam, no. ab182981, 1:1000), anti‐Ki67 (Abcam, no. ab16667, 1:200), anti‐E‐Cadherin (Abcam, no. ab40772, 1:400), anti‐SNAIL+SLUG (Abcam, no. ab85936, 1µg/ml), and anti‐vimentin (Abcam, no. ab92547, 1:200). This was followed by a 30‐min incubation with goat anti‐rabbit secondary antibody (Vector Laboratories, no. BA‐1000, 1:400). The stained slides were either captured using a bright‐field microscope (Nikon, USA) or scanned with the Aperio ScanScope (Leica, Nussloch, Germany). The H‐score for HoxB13, snail, vimentin (in the PC‐3 mouse model), and E‐cadherin ranged from 0 to 300. This score was calculated as the product of the Intensity Score, assigned on a scale from 0 to 3 (0 for negative, 1 for weak positive, 2 for moderate positive, 3 for strong positive), multiplied by the percentage of cells in each tumor sample exhibiting maximum intensity (0–100%). In assessing CD31 and vimentin (in the LNCaP95 mouse model) immunostaining, Microvessel Density (MVD) was appraised in areas of the invasive tumor featuring the highest concentration of capillaries and small venules.^[^ ^66^ ^]^ Regions rich in vascularity were identified at low power (100x), and micro‐vessels were quantified within a high‐power field (400x). MVD was evaluated across three non‐overlapping fields, and the final MVD was determined as the mean. Ki67 was scored by the proliferation index.

Terminal deoxynucleotidyl transferase dUTP nick‐end labeling (TUNEL)assay: A One‐step TUNEL In Situ Apoptosis kit (Elabscience, no. E‐CK‐A321) was used to assess apoptosis in tissue sections, following the manufacturer's protocol.

### Statistical Analysis

All statistical analyses were performed using GraphPad Prism software version 10.0. Data were first evaluated for normality and outliers prior to analysis. Results are presented as mean ± standard deviation (SD) or standard error of the mean (SEM), and the sample size (*n*) for each experiment is indicated in the corresponding figure legends.

For comparisons between two groups, a two‐tailed Student's *t*‐test or the Wald test was used. For comparisons among multiple groups, one‐way ANOVA was employed, followed by appropriate post‐hoc tests when applicable. Survival analysis was performed using the log‐rank (Mantel–Cox) test. Statistical significance was defined as follows: ^*^ *p* < 0.05, ^**^ *p* < 0.01, ^***^ *p* < 0.001, and ^****^ *p* < 0.0001. A significance level of 0.05 was used for all tests.

### Data and Code Availability

The RNA‐seq data generated in this study have been deposited in the Gene Expression Omnibus database under accession number GSE264062.

This paper does not report original code.

Any additional information required to reanalyze the data reported in this paper is available from the lead contact upon request.

## Conflict of Interest

Q.W., Z.C., and Y.D. are inventors on a patent filed by Duke University that relates to the research reported in this paper. J.H. is a consultant for or owns shares in the following companies: Kingmed, MoreHealth, OptraScan, Genetron, Omnitura, Vetonco, York Biotechnology, Genecode, VIVA Biotech, and Sisu Pharma, and received grants from Zenith Epigenetics, BioXcel Therapeutics, Inc., and Fortis Therapeutics.

## Author Contributions

Q.W. conceptualized the project; Q.W., Y.D., Z.C., and H.W. conceptualized and designed the study; Z.C., F.H., J.Y., Y.Z., D.D.K., and W.H. designed and performed experiments; Z.C., K.F., Y.Z., Y.Z., J.I.E., and V.X.J. analyzed the data; A.J.A. and J.H. provided constructive discussion and contributed to editing, Z.C., H.W., Y.D., and Q.W. wrote the manuscript.

## Supporting information



Supporting Information

## Data Availability

The data that support the findings of this study are available from the corresponding author upon reasonable request.

## References

[1] a) C. L. Chaffer , R. A. Weinberg , Science 2011, 331, 1559; 21436443 10.1126/science.1203543

[2] H. Sung , J. Ferlay , R. L. Siegel , M. Laversanne , I. Soerjomataram , A. Jemal , F. Bray , CA Cancer J Clin 2021, 71, 209. 33538338 10.3322/caac.21660

[3] K. Ganesh , J. Massague , Nat. Med. 2021, 27, 34. 33442008 10.1038/s41591-020-01195-4 PMC7895475

[4] J. E. Darnell Jr. , Nat. Rev. Cancer 2002, 2, 740. 12360277 10.1038/nrc906

[5] J. E. Bradner , D. Hnisz , R. A. Young , Cell 2017, 168, 629. 28187285 10.1016/j.cell.2016.12.013 PMC5308559

[6] M. J. Henley , A. N. Koehler , Nat Rev Drug Discov 2021, 20, 669. 34006959 10.1038/s41573-021-00199-0

[7] J. H. Bushweller , Nat. Rev. Cancer 2019, 19, 611. 31511663 10.1038/s41568-019-0196-7 PMC8820243

[8] X. Xie , T. Yu , X. Li , N. Zhang , L. J. Foster , C. Peng , W. Huang , G. He , Signal Transduct Target Ther 2023, 8, 335. 37669923 10.1038/s41392-023-01589-z PMC10480221

[9] a) A. Birmingham , E. M. Anderson , A. Reynolds , D. Ilsley‐Tyree , D. Leake , Y. Fedorov , S. Baskerville , E. Maksimova , K. Robinson , J. Karpilow , W. S. Marshall , A. Khvorova , Nat. Methods 2006, 3, 199; 16489337 10.1038/nmeth854

[10] M. Khoshandam , H. Soltaninejad , M. Mousazadeh , A. A. Hamidieh , S. Hosseinkhani , Genes Dis 2024, 11, 268. 37588217 10.1016/j.gendis.2023.02.027 PMC10425811

[11] S. Konermann , P. Lotfy , N. J. Brideau , J. Oki , M. N. Shokhirev , P. D. Hsu , Cell 2018, 173, 665. 29551272 10.1016/j.cell.2018.02.033 PMC5910255

[12] a) M. Kosicki , K. Tomberg , A. Bradley , Nat. Biotechnol. 2018, 36, 765; 30010673 10.1038/nbt.4192 PMC6390938

[13] a) C. Xu , Y. Zhou , Q. Xiao , B. He , G. Geng , Z. Wang , B. Cao , X. Dong , W. Bai , Y. Wang , X. Wang , D. Zhou , T. Yuan , X. Huo , J. Lai , H. Yang , Nat. Methods 2021, 18, 499; 33941935 10.1038/s41592-021-01124-4

[14] a) J. Eoh , L. Gu , Biomater. Sci. 2019, 7, 1240; 30734775 10.1039/c8bm01310a

[15] a) H. Zhang , X. You , X. Wang , L. Cui , Z. Wang , F. Xu , M. Li , Z. Yang , J. Liu , P. Huang , Y. Kang , J. Wu , X. Xia , Proc. Natl. Acad. Sci. USA 2021, 118; 10.1073/pnas.2005191118 PMC8017939 33547233

[16] D. I. Tsilimigras , P. Brodt , P. A. Clavien , R. J. Muschel , M. I. D'Angelica , I. Endo , R. W. Parks , M. Doyle , E. de Santibanes , T. M. Pawlik , Nat. Rev. Dis. Primers 2021, 7, 27. 33859205 10.1038/s41572-021-00261-6

[17] K. T. Love , K. P. Mahon , C. G. Levins , K. A. Whitehead , W. Querbes , J. R. Dorkin , J. Qin , W. Cantley , L. L. Qin , T. Racie , M. Frank‐Kamenetsky , K. N. Yip , R. Alvarez , D. W. Sah , A. de Fougerolles , K. Fitzgerald , V. Koteliansky , A. Akinc , R. Langer , D. G. Anderson , Proc Natl Acad Sci U S A 2010, 107, 1864. 20080679 10.1073/pnas.0910603106 PMC2804742

[18] a) L. Bubendorf , A. Schopfer , U. Wagner , G. Sauter , H. Moch , N. Willi , T. C. Gasser , M. J. Mihatsch , Hum Pathol 2000, 31, 578; 10836297 10.1053/hp.2000.6698

[19] a) G. R. Pond , G. Sonpavde , R. de Wit , M. A. Eisenberger , I. F. Tannock , A. J. Armstrong , Eur Urol 2014, 65, 3; 24120464 10.1016/j.eururo.2013.09.024

[20] a) Z. Chen , D. Wu , J. M. Thomas‐Ahner , C. Lu , P. Zhao , Q. Zhang , C. Geraghty , P. S. Yan , W. Hankey , B. Sunkel , X. Cheng , E. S. Antonarakis , Q. E. Wang , Z. Liu , T. H. Huang , V. X. Jin , S. K. Clinton , J. Luo , J. Huang , Q. Wang , Proc Natl Acad Sci U S A 2018, 115, 6810; 29844167 10.1073/pnas.1718811115 PMC6042123

[21] M. Yu , J. Zhan , H. Zhang , Cell Signal 2020, 66, 109469. 31733300 10.1016/j.cellsig.2019.109469

[22] D. B. T. Cox , J. S. Gootenberg , O. O. Abudayyeh , B. Franklin , M. J. Kellner , J. Joung , F. Zhang , Science 2017, 358, 1019. 29070703 10.1126/science.aaq0180 PMC5793859

[23] a) K. J. Kron , A. Murison , S. Zhou , V. Huang , T. N. Yamaguchi , Y. J. Shiah , M. Fraser , T. van der Kwast , P. C. Boutros , R. G. Bristow , M. Lupien , Nat. Genet. 2017, 49, 1336; 28783165 10.1038/ng.3930

[24] F. Tang , D. Xu , S. Wang , C. K. Wong , A. Martinez‐Fundichely , C. J. Lee , S. Cohen , J. Park , C. E. Hill , K. Eng , R. Bareja , T. Han , E. M. Liu , A. Palladino , W. Di , D. Gao , W. Abida , S. Beg , L. Puca , M. Meneses , E. de Stanchina , M. F. Berger , A. Gopalan , L. E. Dow , J. M. Mosquera , H. Beltran , C. N. Sternberg , P. Chi , H. I. Scher , A. Sboner , et al., Science 2022, 376, abe1505. 10.1126/science.abe1505 PMC9299269 35617398

[25] a) B. P Gray , L. Kelly , D. P. Ahrens , A. P. Barry , C. Kratschmer , M. Levy , B. A. Sullenger , Proc Natl Acad Sci U S A 2018, 115, 4761; 29666232 10.1073/pnas.1717705115 PMC5939073

[26] B. P Gray , X. Song , D. S. Hsu , C. Kratschmer , M. Levy , A. P. Barry , B. A. Sullenger , Cancers (Basel) 2020, 12. 33142831 10.3390/cancers12113217 PMC7694147

[27] M. Kim , M. Jeong , S. Hur , Y. Cho , J. Park , H. Jung , Y. Seo , H. A. Woo , K. T. Nam , K. Lee , H. Lee , Sci. Adv. 2021, 7. 10.1126/sciadv.abf4398 PMC7909888 33637537

[28] F. Braet , E. Wisse , Comp. Hepatol. 2002, 1, 1. 12437787 10.1186/1476-5926-1-1 PMC131011

[29] a) V. P. Chauhan , R. K. Jain , Nat. Mater. 2013, 12, 958; 24150413 10.1038/nmat3792 PMC4120281

[30] a) S. C. Semple , A. Akinc , J. Chen , A. P. Sandhu , B. L. Mui , C. K. Cho , D. W. Sah , D. Stebbing , E. J. Crosley , E. Yaworski , I. M. Hafez , J. R. Dorkin , J. Qin , K. Lam , K. G. Rajeev , K. F. Wong , L. B. Jeffs , L. Nechev , M. L. Eisenhardt , M. Jayaraman , M. Kazem , M. A. Maier , M. Srinivasulu , M. J. Weinstein , Q. Chen , R. Alvarez , S. A. Barros , S. De , S. K. Klimuk , T. Borland , et al., Nat. Biotechnol. 2010, 28, 172; 20081866 10.1038/nbt.1602

[31] M. Danaei , M. Dehghankhold , S. Ataei , F. Hasanzadeh Davarani , R. Javanmard , A. Dokhani , S. Khorasani , M. R. Mozafari , Pharmaceutics 2018, 10. 10.3390/pharmaceutics10020057 PMC6027495 29783687

[32] B. W. Simons , S. Dalrymple , M. Rosen , L. Zheng , W. N. Brennen , Prostate 2020, 80, 1263. 32761950 10.1002/pros.24055

[33] a) D. L. Wagner , L. Amini , D. J. Wendering , L. M. Burkhardt , L. Akyuz , P. Reinke , H. D. Volk , M. Schmueck‐Henneresse , Nat. Med. 2019, 25, 242; 30374197 10.1038/s41591-018-0204-6

[34] F. Palaz , A. K. Kalkan , O. Can , A. N. Demir , A. Tozluyurt , A. Ozcan , M. Ozsoz , ACS Synth. Biol. 2021, 10, 1245. 34037380 10.1021/acssynbio.1c00107

[35] M. C. Paul , C. Schneeweis , C. Falcomata , C. Shan , D. Rossmeisl , S. Koutsouli , C. Klement , M. Zukowska , S. A. Widholz , M. Jesinghaus , K. K. Heuermann , T. Engleitner , B. Seidler , K. Sleiman , K. Steiger , M. Tschurtschenthaler , B. Walter , S. A. Weidemann , R. Pietsch , A. Schnieke , R. M. Schmid , M. S. Robles , G. Andrieux , M. Boerries , R. Rad , G. Schneider , D. Saur , Nat. Commun. 2023, 14, 1201. 36882420 10.1038/s41467-023-36505-0 PMC9992512

[36] J. Yan , M. Enge , T. Whitington , K. Dave , J. Liu , I. Sur , B. Schmierer , A. Jolma , T. Kivioja , M. Taipale , J. Taipale , Cell 2013, 154, 801. 23953112 10.1016/j.cell.2013.07.034

[37] R. Zheng , C. Wan , S. Mei , Q. Qin , Q. Wu , H. Sun , C. H. Chen , M. Brown , X. Zhang , C. A. Meyer , X. S. Liu , Nucleic Acids Res. 2019, 47, D729. 30462313 10.1093/nar/gky1094 PMC6324081

[38] S. Li , C. Wan , R. Zheng , J. Fan , X. Dong , C. A. Meyer , X. S. Liu , Nucleic Acids Res. 2019, 47, W206. 31053864 10.1093/nar/gkz332 PMC6602521

[39] a) K. F. Harvey , X. Zhang , D. M. Thomas , Nat. Rev. Cancer 2013, 13, 246; 23467301 10.1038/nrc3458

[40] a) S. Telang , A. L. Clem , J. W. Eaton , J. Chesney , Neoplasia 2007, 9, 47; 17325743 10.1593/neo.06664 PMC1804324

[41] T. Mitamura , S. Pradeep , M. McGuire , S. Y. Wu , S. Ma , H. Hatakeyama , Y. A. Lyons , T. Hisamatsu , K. Noh , A. Villar‐Prados , X. Chen , C. Ivan , C. Rodriguez‐Aguayo , W. Hu , G. Lopez‐Berestein , R. L. Coleman , A. K. Sood , Oncogene 2018, 37, 722. 29059175 10.1038/onc.2017.348 PMC6040890

[42] A. Zhang , J. Zhang , S. Plymate , E. A. Mostaghel , Horm Cancer 2016, 7, 104. 26797685 10.1007/s12672-016-0250-9 PMC4859429

[43] H. Li , N. Xie , R. Chen , M. Verreault , L. Fazli , M. E. Gleave , O. Barbier , X. Dong , Cancer Res. 2016, 76, 6701. 27659047 10.1158/0008-5472.CAN-16-1518

[44] D. Hanahan , Cancer Discov 2022, 12, 31. 35022204 10.1158/2159-8290.CD-21-1059

[45] L. Hood , J. R. Heath , M. E. Phelps , B. Lin , Science 2004, 306, 640. 15499008 10.1126/science.1104635

[46] a) P. Colella , G. Ronzitti , F. Mingozzi , Mol Ther Methods Clin Dev 2018, 8, 87; 29326962 10.1016/j.omtm.2017.11.007 PMC5758940

[47] X. Hou , T. Zaks , R. Langer , Y. Dong , Nat. Rev. Mater. 2021, 6, 1078. 34394960 10.1038/s41578-021-00358-0 PMC8353930

[48] a) A. Akinc , M. A. Maier , M. Manoharan , K. Fitzgerald , M. Jayaraman , S. Barros , S. Ansell , X. Du , M. J. Hope , T. D. Madden , B. L. Mui , S. C. Semple , Y. K. Tam , M. Ciufolini , D. Witzigmann , J. A. Kulkarni , R. van der Meel , P. R. Cullis , Nat. Nanotechnol. 2019, 14, 1084; 31802031 10.1038/s41565-019-0591-y

[49] J. H. Wang , D. J. Gessler , W. Zhan , T. L. Gallagher , G. Gao , Signal Transduct Target Ther 2024, 9, 78. 38565561 10.1038/s41392-024-01780-w PMC10987683

[50] Q. Cheng , T. Wei , L. Farbiak , L. T. Johnson , S. A. Dilliard , D. J. Siegwart , Nat. Nanotechnol. 2020, 15, 313. 32251383 10.1038/s41565-020-0669-6 PMC7735425

[51] N. K. Altorki , G. J. Markowitz , D. Gao , J. L. Port , A. Saxena , B. Stiles , T. McGraw , V. Mittal , Nat. Rev. Cancer 2019, 19, 9. 30532012 10.1038/s41568-018-0081-9 PMC6749995

[52] A. Cano , M. A. Perez‐Moreno , I. Rodrigo , A. Locascio , M. J. Blanco , M. G. del Barrio , F. Portillo , M. A. Nieto , Nat. Cell Biol. 2000, 2, 76. 10655586 10.1038/35000025

[53] Q. Wang , X. Liu , J. Zhou , C. Yang , G. Wang , Y. Tan , Y. Wu , S. Zhang , K. Yi , C. Kang , Adv. Sci. (Weinh) 2019, 6, 1901299. 31637166 10.1002/advs.201901299 PMC6794629

[54] P. Bule , V. D. Alves , A. Leitao , L. M. Ferreira , E. A. Bayer , S. P. Smith , H. J. Gilbert , S. Najmudin , C. M. Fontes , J. Biol. Chem. 2016, 291, 26658. 27875311 10.1074/jbc.M116.761643 PMC5207176

[55] a) B. Li , X. Luo , Y. Dong , Bioconjug Chem 2016, 27, 849; 26906521 10.1021/acs.bioconjchem.6b00090

[56] K. C. Valkenburg , S. R. Amend , K. J. Pienta , J Vis Exp 2016.

[57] Y. Xu , L. Zhang , J. Thaiparambil , S. Mai , D. N. Perera , J. Zhang , P. Y. Pan , C. Coarfa , K. Ramos , S. H. Chen , R. El‐Zein , Cancer Res Commun 2022, 2, 884. 36923308 10.1158/2767-9764.CRC-22-0057 PMC10010305

[58] S. Berg , D. Kutra , T. Kroeger , C. N. Straehle , B. X. Kausler , C. Haubold , M. Schiegg , J. Ales , T. Beier , M. Rudy , K. Eren , J. I. Cervantes , B. Xu , F. Beuttenmueller , A. Wolny , C. Zhang , U. Koethe , F. A. Hamprecht , A. Kreshuk , Nat. Methods 2019, 16, 1226. 31570887 10.1038/s41592-019-0582-9

[59] D. R. Stirling , M. J. Swain‐Bowden , A. M. Lucas , A. E. Carpenter , B. A. Cimini , A. Goodman , BMC Bioinformatics 2021, 22, 433. 34507520 10.1186/s12859-021-04344-9 PMC8431850

[60] D. Schapiro , H. W. Jackson , S. Raghuraman , J. R. Fischer , V. R. T. Zanotelli , D. Schulz , C. Giesen , R. Catena , Z. Varga , B. Bodenmiller , Nat. Methods 2017, 14, 873. 28783155 10.1038/nmeth.4391 PMC5617107

[61] M. I. Love , W. Huber , S. Anders , Genome Biol. 2014, 15, 550. 25516281 10.1186/s13059-014-0550-8 PMC4302049

[62] T. Galore , https://www.bioinformatics.babraham.ac.uk/projects/trim_galore/

[63] D. Kim , J. M. Paggi , C. Park , C. Bennett , S. L. Salzberg , Nat. Biotechnol. 2019, 37, 907. 31375807 10.1038/s41587-019-0201-4 PMC7605509

[64] Y. Liao , G. K. Smyth , W. Shi , Nucleic Acids Res. 2013, 41, 108. 10.1093/nar/gkt214 PMC3664803 23558742

[65] A. R. Quinlan , I. M. Hall , Bioinformatics 2010, 26, 841. 20110278 10.1093/bioinformatics/btq033 PMC2832824

[66] a) N. Weidner , J. P. Semple , W. R. Welch , J. Folkman , N. Engl. J. Med. 1991, 324, 1; 10.1056/NEJM199101033240101 1701519

