# Supplementary material for: SCORT–Cas13d Nanotherapy Precisely Targets the ‘Undruggable’ Transcription Factor HoxB13 in Metastatic Prostate Cancer In Vivo
Source: Adv Sci (Weinh). 2025 May 11;12(23):2417605. doi: 10.1002/advs.202417605 (PMC12199340; doi:10.1002/advs.202417605)
Supplement: Supplementary file 1 — Supporting Information [file ADVS-12-2417605-s001.pdf]

ADVANCED SCIENCE Open Access

Supporting Information

for *Adv. Sci.*, DOI 10.1002/advs.202417605

SCORT–Cas13d Nanotherapy Precisely Targets the 'Undruggable' Transcription Factor HoxB13 in Metastatic Prostate Cancer In Vivo

Zhifen Cui, Furong Huang, Kun Fang, Jingyue Yan, Yuebao Zhang, Diana D. Kang, Yufan Zhou, Yue Zhao, Jeffrey I. Everitt, William Hankey, Andrew J Armstrong, Jiaoti Huang, Hongyan Wang, Victor X. Jin, Yizhou Dong and Qianben Wang**

SCORT–Cas13d Nanotherapy Precisely Targets the 'Undruggable' Transcription Factor HoxB13 in Metastatic Prostate Cancer In Vivo

Zhifen Cui, Furong Huang, Kun Fang, Jingyue Yan, Yuebao Zhang, Diana D. Kang, Yufan Zhou, Yue Zhao, Jeffrey I. Everitt, William Hankey, Andrew J Armstrong, Jiaoti Huang, Hongyan Wang, Victor X. Jin, Yizhou Dong, and Qianben Wang

Z. Cui, F. Huang, Y. Zhao, J. Everitt, W. Hankey, J. Huang, H. Wang, Q. Wang
Department of Pathology
Duke University School of Medicine
Durham, NC 27710, USA
E-mail: qianben.wang@duke.edu
K. Fang, V. Jin
Medical College of Wisconsin Cancer Center
Medical College of Wisconsin,
8701 Watertown Plank Road, Milwaukee, WI 53226, USA
J. Yan, Y. Zhang, D. Kang
Division of Pharmaceutics & Pharmacology
College of Pharmacy
The Ohio State University
Columbus, OH 43210, USA
D. Kang, Y. Dong
Icahn Genomics Institute
Precision Immunology Institute
Department of Immunology and Immunotherapy
Department of Oncological Sciences
Tisch Cancer Institute
Biomedical Engineering and Imaging Institute
Friedman Brain Institute
Icahn School of Medicine at Mount Sinai
New York, NY, 10029, USA
E-mail: yizhou.dong@mssm.edu
Y. Zhou
Department of Biochemistry and Structural Biology
University of Texas Health San Antonio
San Antonio, TX 78229, USA
A. Armstrong
Department of Medicine
Duke University School of Medicine

Durham, NC 27710, USA
A. Armstrong, Q. Wang
Duke Cancer Institute Center for Prostate and Urologic Cancer
Durham, NC 27710, USA
Q. Wang
Department of Cell Biology
Duke University School of Medicine
Durham, NC 27710, USA

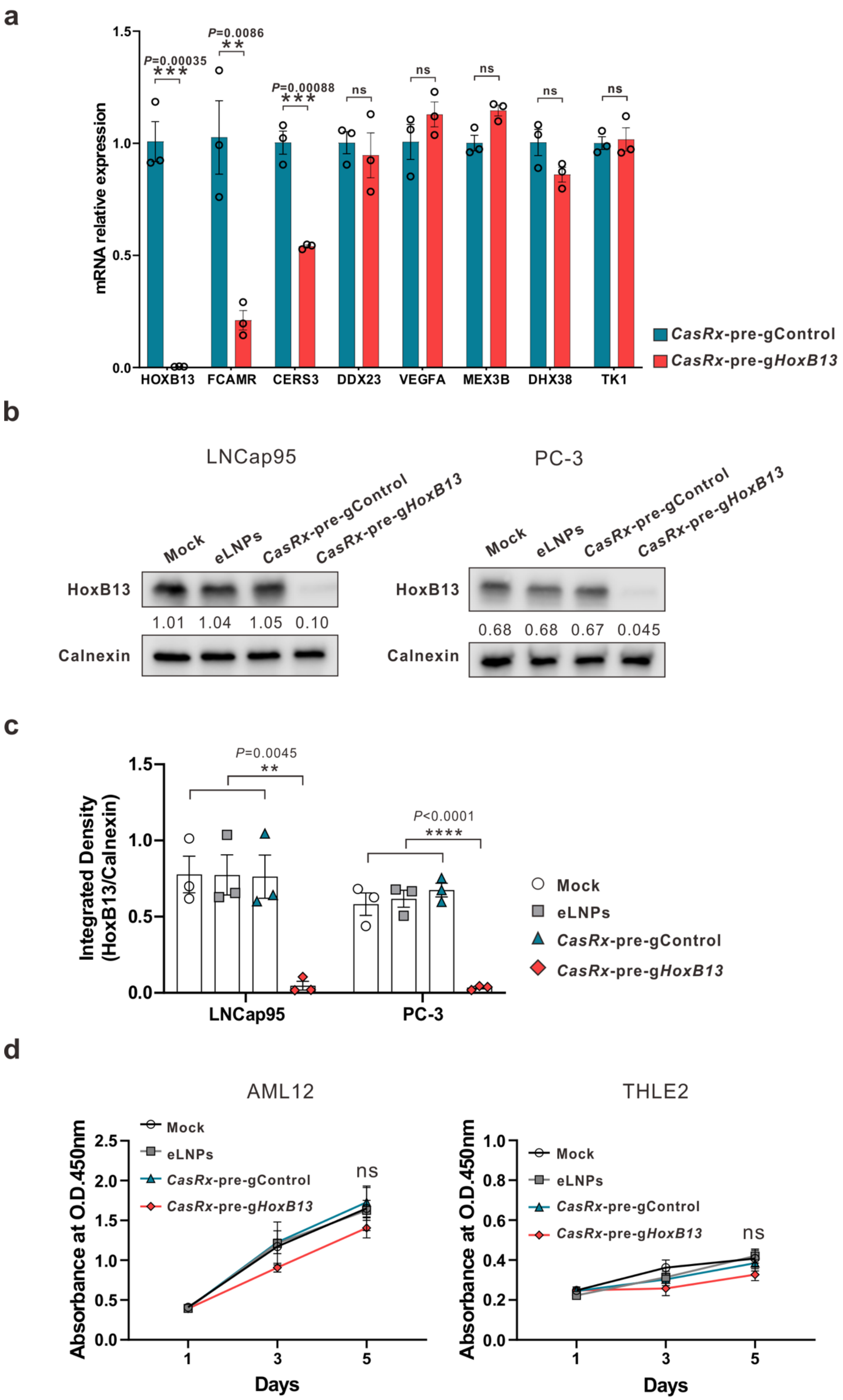
a
mRNA relative expression
P=0.00035
***
P=0.0086
**
P=0.00088
***
ns
HOXB13
FCAMR
CERS3
DDX23
VEGFA
MEX3B
DHX38
TK1
CasRx-pre-gControl
CasRx-pre-gHoxB13
b
LNCap95
PC-3
Mock
eLNPs
CasRx-pre-gControl
CasRx-pre-gHoxB13
HoxB13
Calnexin
1.01 1.04 1.05 0.10
0.68 0.68 0.67 0.045
c
Integrated Density (HoxB13/Calnexin)
P=0.0045
**
P<0.0001
****
LNCap95
PC-3
Mock
eLNPs
CasRx-pre-gControl
CasRx-pre-gHoxB13
d
AML12
THLE2
Absorbance at O.D.450nm
Days
Mock
eLNPs
CasRx-pre-gControl
CasRx-pre-gHoxB13
ns

Figure S1. *CasRx*-pre-g*HoxB13* specifically and effectively reduces HoxB13 protein levels in both AR+ and AR- CRPC cells without causing cytotoxicity in normal cells. a, Transcript levels were normalized to 18S rRNA in 293FT cells. Data are presented as mean ± SEM with n=3 biologically independent replicates, *P* values were calculated by two-tailed Student's *t*-tests. ns, not significant, ***P*<0.01, ****P*<0.001. **b,** *CasRx*-pre-g*HoxB13* reduces HoxB13 protein in LNCaP95 cells (left) and PC-3 cells (right). Samples were similarly treated as those in Fig. 1f-h. The ratio of HoxB13 to the loading control calnexin is listed below the blot. **c**, Integrated density of HoxB13 normalized to calnexin as a quantification measure. Data are presented as mean ± SEM with n=3 biologically independent replicates. **d**, No significant cytotoxicity of *CasRx*-pre-g*HoxB13* in normal liver cells. Data are presented as mean ± SD with n=5 biologically independent replicates. *P* values for c and d were calculated by One-way ANOVA. ns, not significant, ***P*<0.01, *****P*<0.0001.

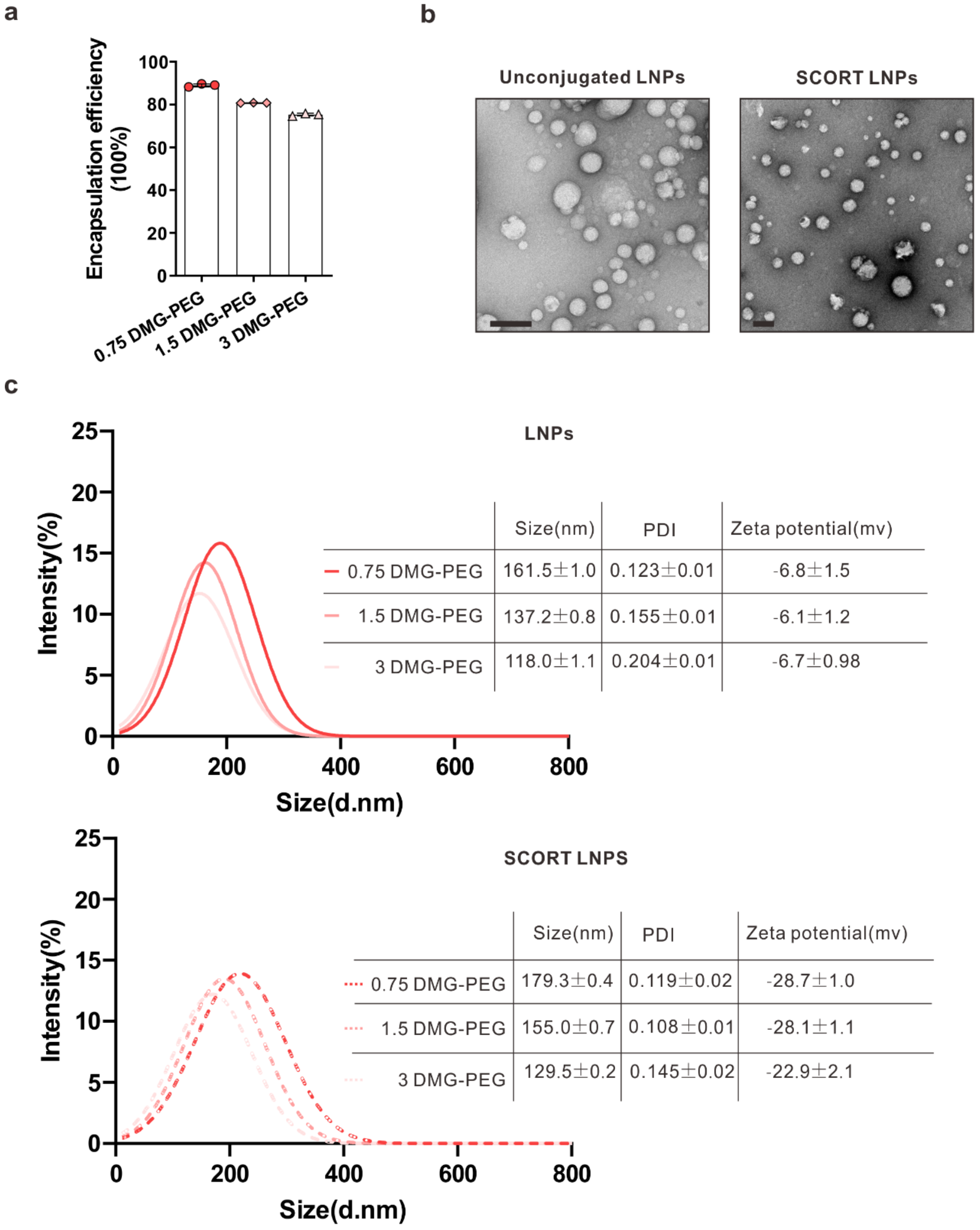

Figure S2. Characterization of LNPs and SCORT LNPs. a, The encapsulation efficiency of LNPs candidates was determined by RiboGreen assay in triplicates. **b**, Representative transmission electron microscopy (TEM) images of LNPs and SCORT LNPs. Scale bar: 200 nm. **c**, Size distribution, polydispersity index (PDI), and zeta potential of LNPs and SCORT LNPs candidates. Data are presented as mean ± SD.

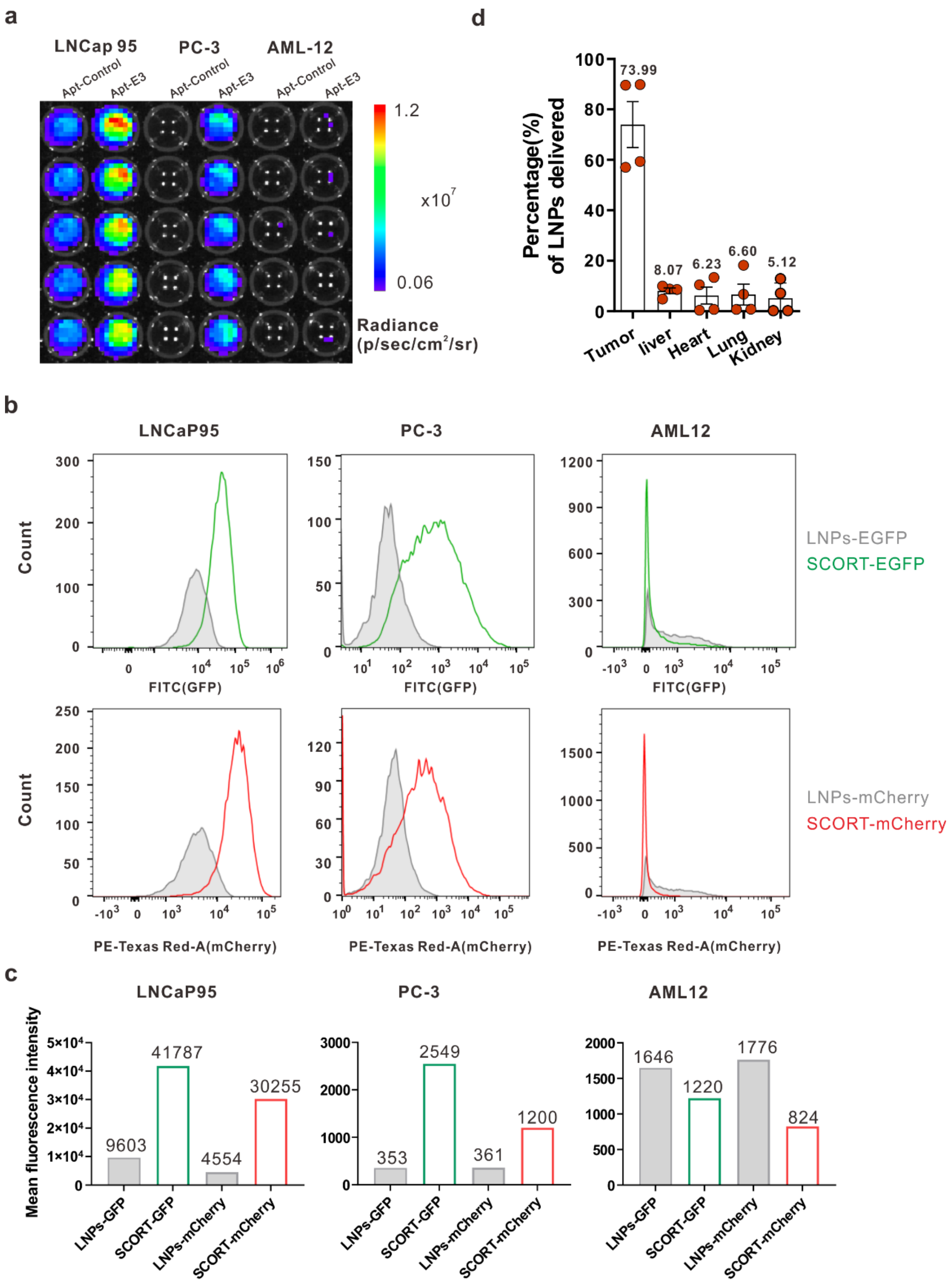

Figure S3. SCORT LNPs selectively deliver mRNA to AR$^+$ and AR$^-$ CRPC cells while sparing normal liver and other major organs. a, Luminescence imaging of LNCaP95, PC-3 and AML12 cells after 24 h incubation with *luciferase* mRNA-encapsulated LNPs or SCORT LNPs (3 DMG-PEG for LNCaP95; 0.75 DMG-PEG for PC-3 and AML12). **b,** Flow cytometry analyses of LNCaP95, PC-3, and AML12 cells following 24 h incubation with *EGFP* or *mCherry* mRNA-encapsulated LNPs or SCORT LNPs. **c**, Mean fluorescence intensity of flow cytometry analysis. **d**, Percentages of LNPs delivered to major organs, including liver, heart, lungs, and kidneys, as well as the tumor, each dot represents an individual mouse (n=4).

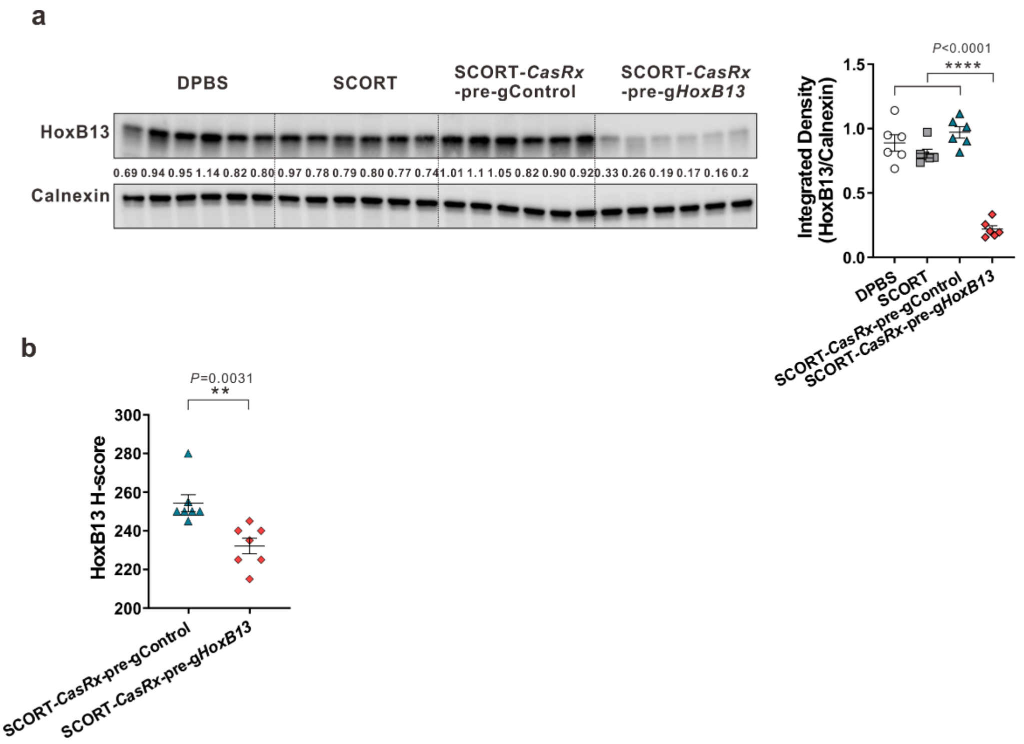

Figure S4. SCORT-*CasRx*-pre-g*HoxB13* treatment reduces HoxB13 protein level in liver metastatic tumors. a, Western blot of HoxB13 protein. Each lane represents an individual mouse (n=6). The ratio of HoxB13 to the loading control calnexin is listed below the blot (left). Integrated density of HoxB13 normalized to calnexin is shown as a quantification measure (right). Data are presented as mean ± SEM. *P* values were calculated by one-way ANOVA. ****P<0.0001. **b**, H-score for HoxB13 immunostaining in Fig. 3f (n=7). Data are presented as mean ± SEM. *P* values were calculated by two-tailed Student's *t*-test. **P<0.01.

a

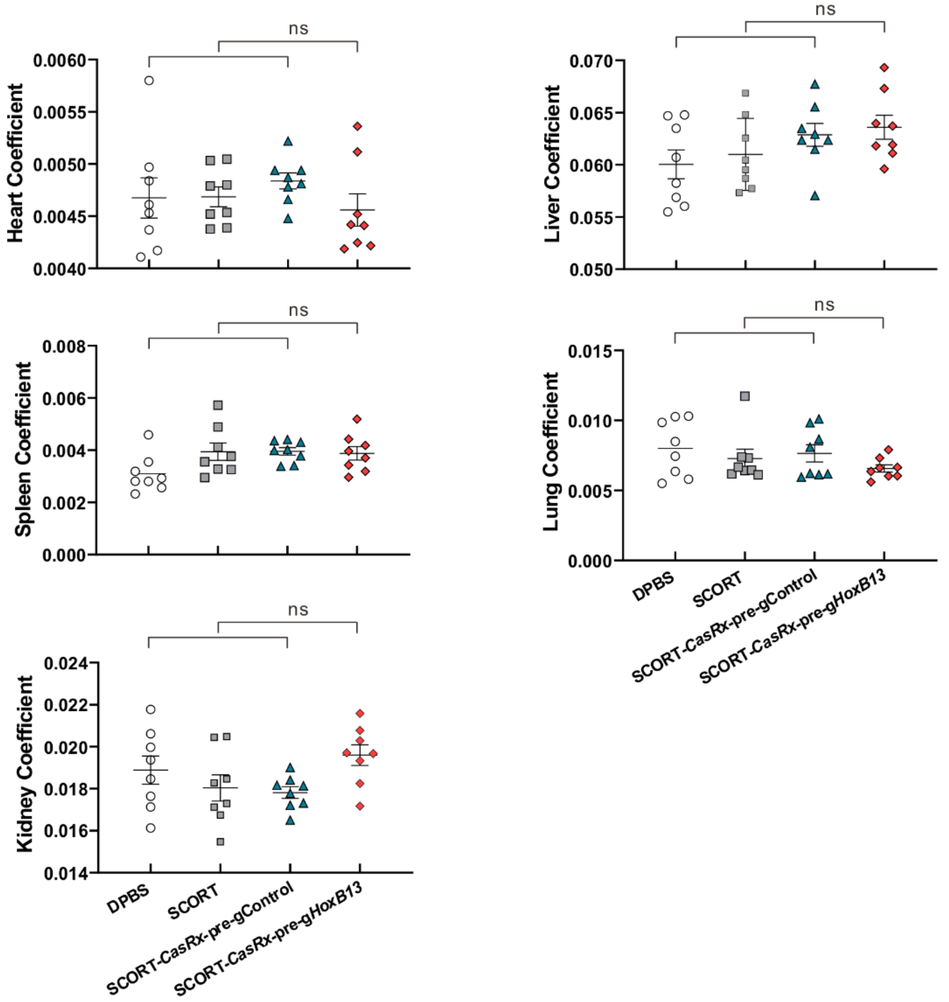

Heart Coefficient
Liver Coefficient
Spleen Coefficient
Lung Coefficient
Kidney Coefficient
ns
DPBS
SCORT
SCORT-CasRx-pre-gControl
SCORT-CasRx-pre-gHoxB13

b

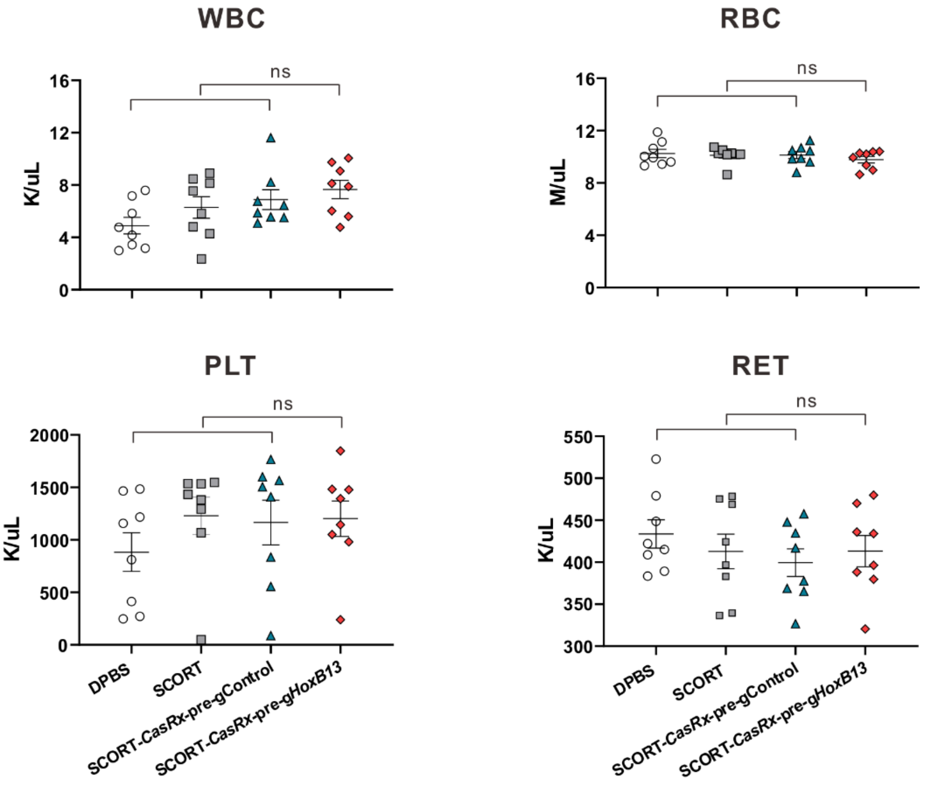

WBC
RBC
PLT
RET
K/uL
M/uL
ns
DPBS
SCORT
SCORT-CasRx-pre-gControl
SCORT-CasRx-pre-gHoxB13

Figure S5. Organ coefficient and hematology evaluation of SCORT-*CasRx*-pre-g*HoxB13* treatment in CD-1 mice (n=8). a, Organ coefficient. The treatments are same as in Fig. 5. Coefficient is the organ weight normalized by the body weight. **b**, Hematology was not impaired by SCORT-*CasRx*-pre-g*HoxB13* treatment. WBC, white blood cell; RBC, red blood cell; PLT, platelet; RET, reticulocyte. All data are presented as mean ± SEM (n=8). *P* values were calculated by One-way ANOVA, ns, not significant.

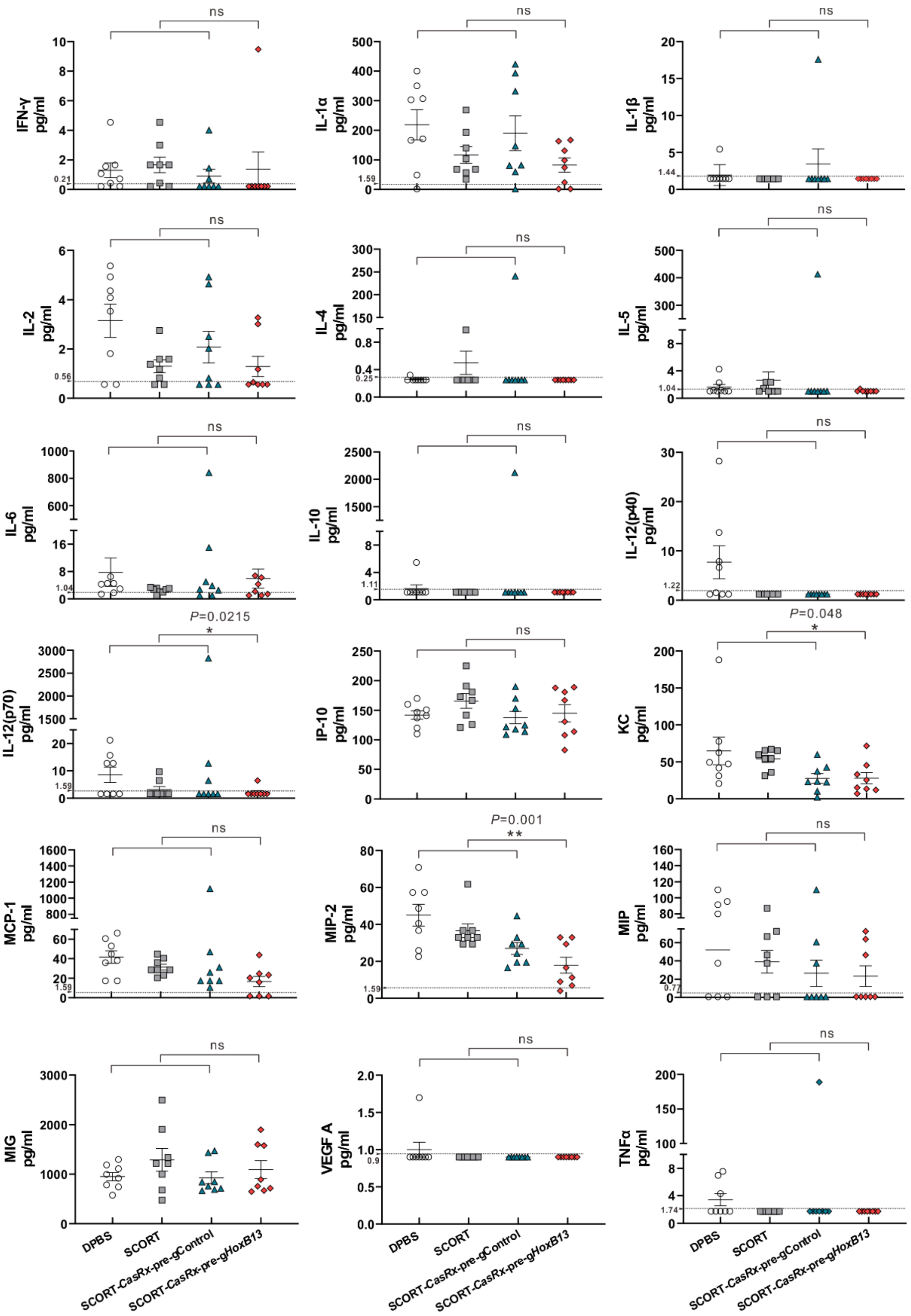

Figure S6. Plasma level of cytokine and chemokines following SCORT-*CasRx*-pre-g*HoxB13* treatment in CD-1 mice (n=8). The treatments are same as in Fig. 5. Data are represented as the mean ± SEM. *P* values were calculated by one-way ANOVA, *$P<0.05$, **$P<0.01$, ns, not significant.

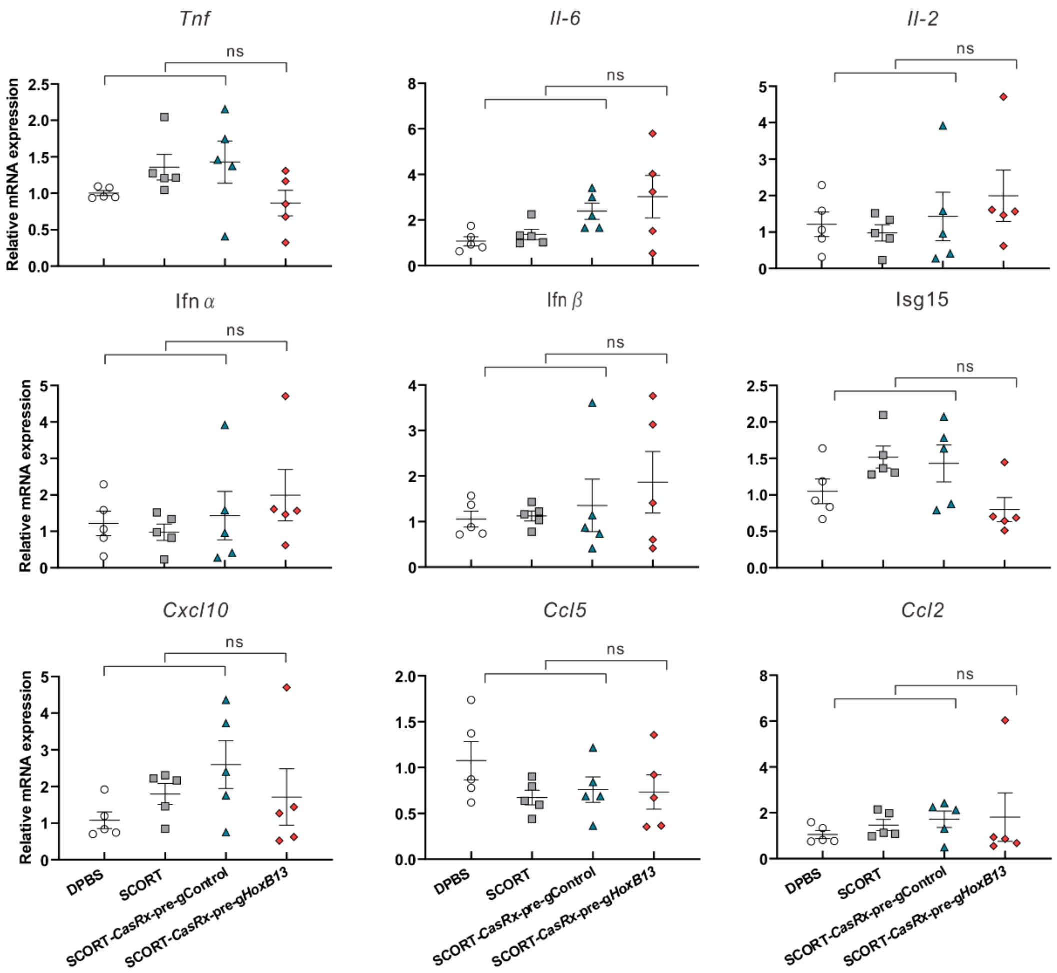

Figure S7. Transcript levels of cytokines and chemokines in the liver of CD-1 mice (n=5) following SCORT-*CasRx*-pre-g*HoxB13* treatment. The treatments are same as in Fig. 5. Data are presented as mean ± SEM. *P* values were calculated using one-way ANOVA. ns, not significant.

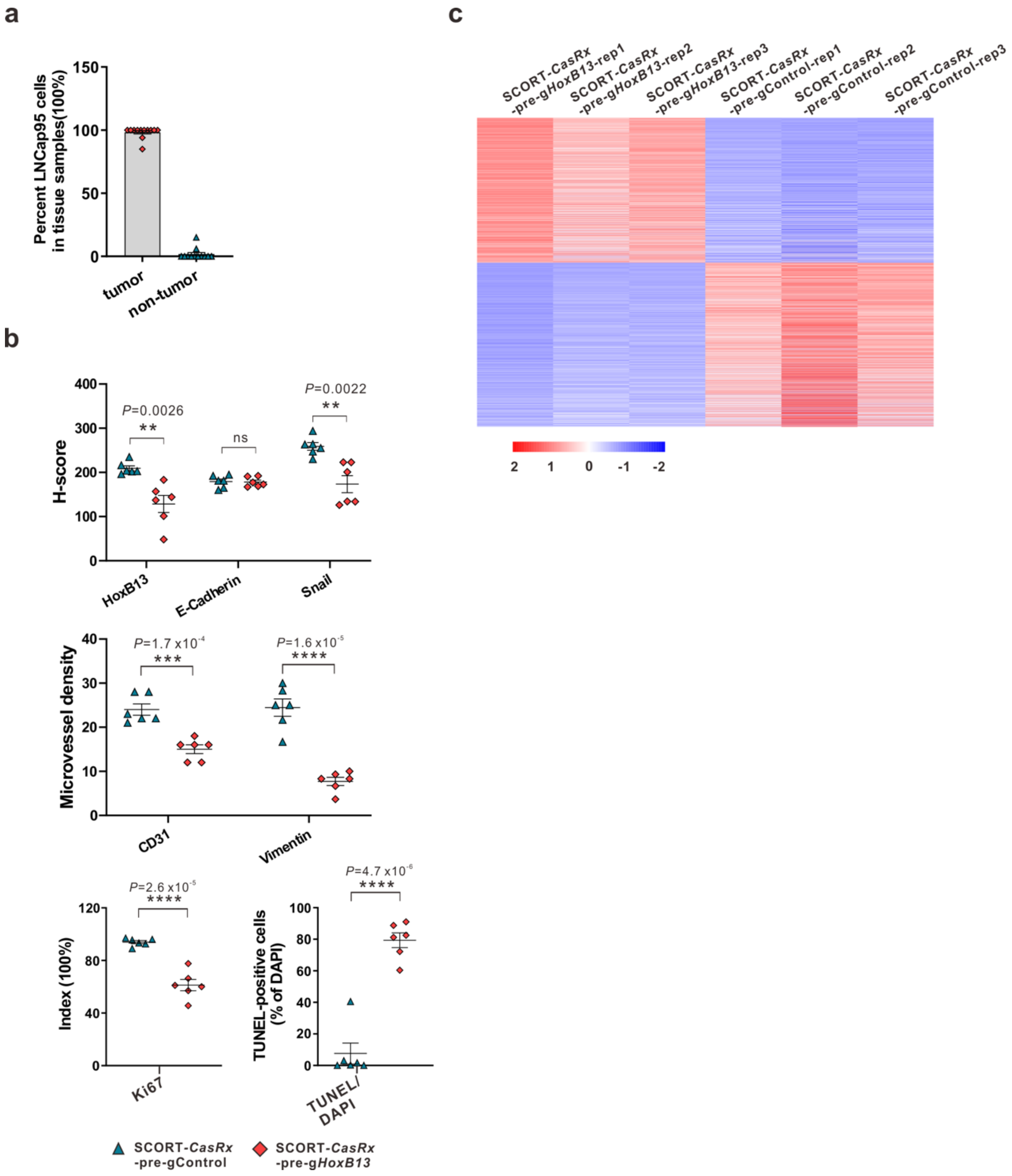

Figure S8. Cellular composition, histological changes, and transcriptional alterations induced by SCORT-*CasRx*-pre-g*HoxB13* in the LNCaP95 liver metastasis model. a, Percentage of LNCaP95 cells within each tumor or non-tumor tissue sections as determined by H&E staining (n=12). **b**, Quantification of immunostaining and TUNEL assay (n=6): H-score for HoxB13, E-Cadherin and Snail; index for Ki67 and micro vessel density indicated by CD31 and vimentin. Data are presented as mean ± SEM. *P* values were calculated by two tailed Student's *t*-tests, $^{**}P<0.01$, $^{***}P<0.001$, $^{****}P<0.0001$. **c**, Heatmap of z-scored expression values of the identified DEGs, with upregulated genes in red and downregulated genes in blue.

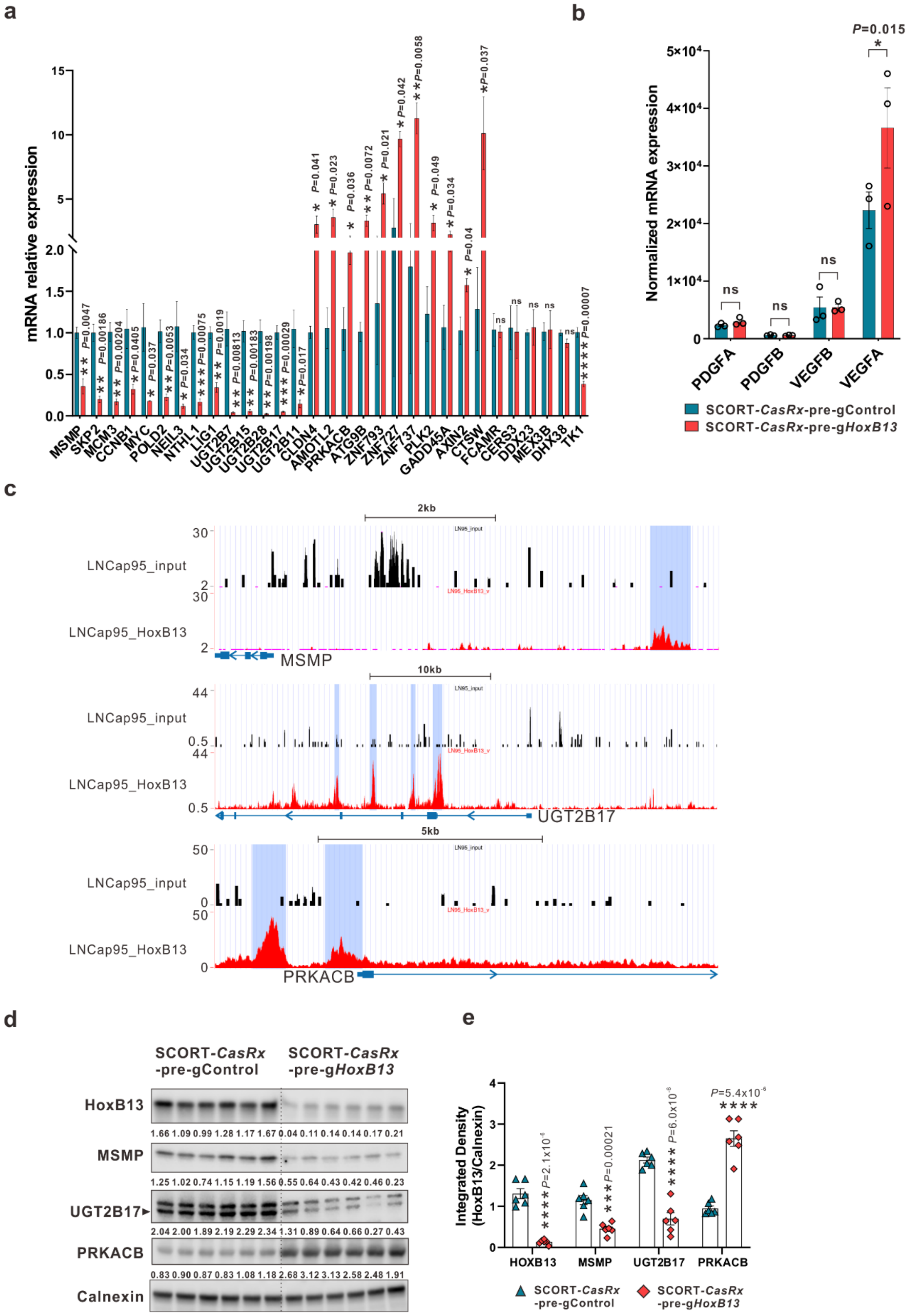

Figure S9. Validation of transcriptional targets and pathway genes following SCORT-*CasRx*-pre-g*HoxB13* treatment in the LNCaP95 liver metastasis model. a, Validation of top-ranked KEGG pathway genes and genes used in 293FT cell–based RNA-seq off-target analysis in liver metastatic tumor samples by qRT-PCR (n=3). **b,** mRNA expression levels of PDGFs and VEGFs. Data are presented as mean ± SEM,

and *P* value were calculated using the Wald test from DESeq2. **P*<0.05, ns, not significant. These genes are not directly regulated by HoxB13, as our LNCaP95 HoxB13 ChIP-exo analysis showed that no HoxB13 binding sites are present within ±20 Kb of their transcription initiation sites. **c,** UCSC Genome Browser visualization of HoxB13 binding peaks near *MSMA*, *UGT2B17* and *PRKACB*. **d**, Western blot of HoxB13, MSMP, UGT2B17 and PRKACB protein. Each lane represents an individual mouse (n=6). The ratio of the proteins to the loading control calnexin is listed below the blot. **e**, Integrated density of the proteins over the loading control for d. Data for a, b, and e are represented the mean ± SEM. *P* values were calculated by two tailed Student's *t*-tests, ns, not significant, **P*<0.05, ***P*<0.01, ****P*<0.001, *****P*<0.0001.

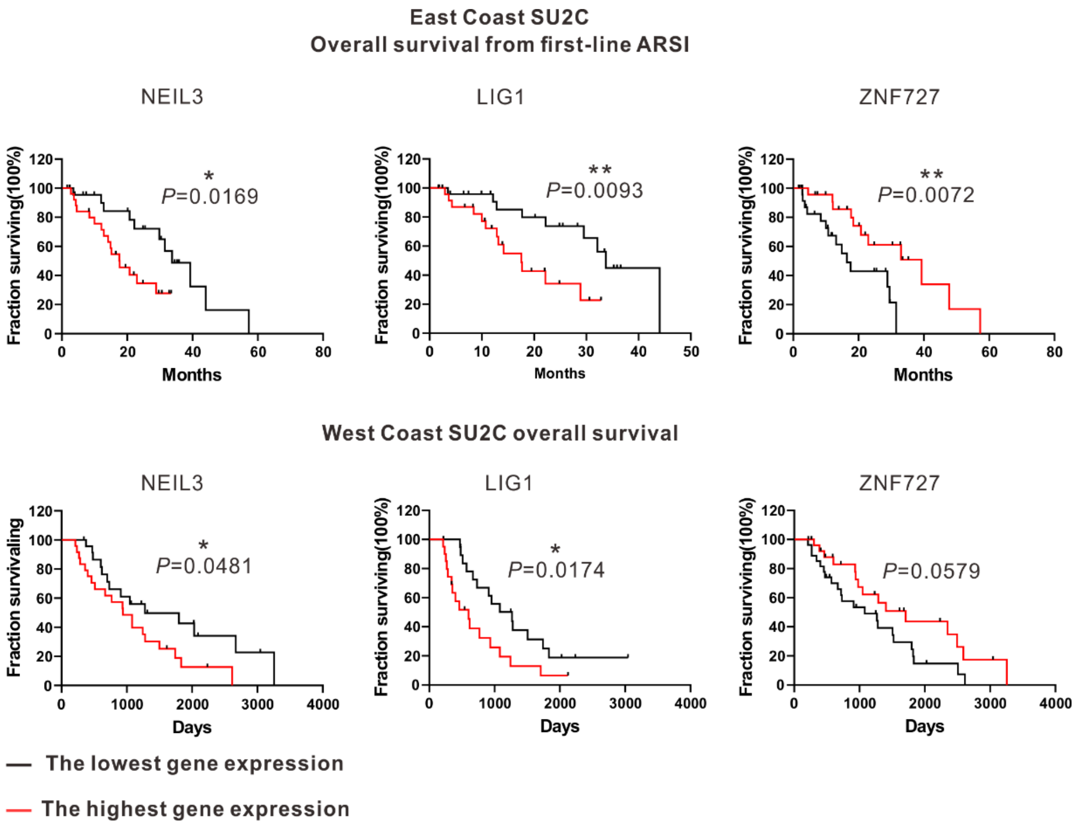

Figure S10. Correlation of gene expression levels with overall survival in SU2C East Coast and West Coast CRPC patient datasets. Upper panels: Overall survival from the start of first line ARSI treatment was compared between the highest and lowest tertile (25 cases) of *NEIL3*, *LIG1*, and *ZNF727* expression within CRPC tumors from the East Coast SU2C dataset. Lower panels: Overall survival was compared between quartiles of *NEIL3* (24 cases), approximate quintiles of *LIG1* (20 cases), and approximate quartiles of *ZNF727* (27 cases) expression in CRPC tumors from the West Coast SU2C dataset. *P*-values were calculated by log-rank (Mantel-Cox) test, **P*<0.05, ***P*<0.01.

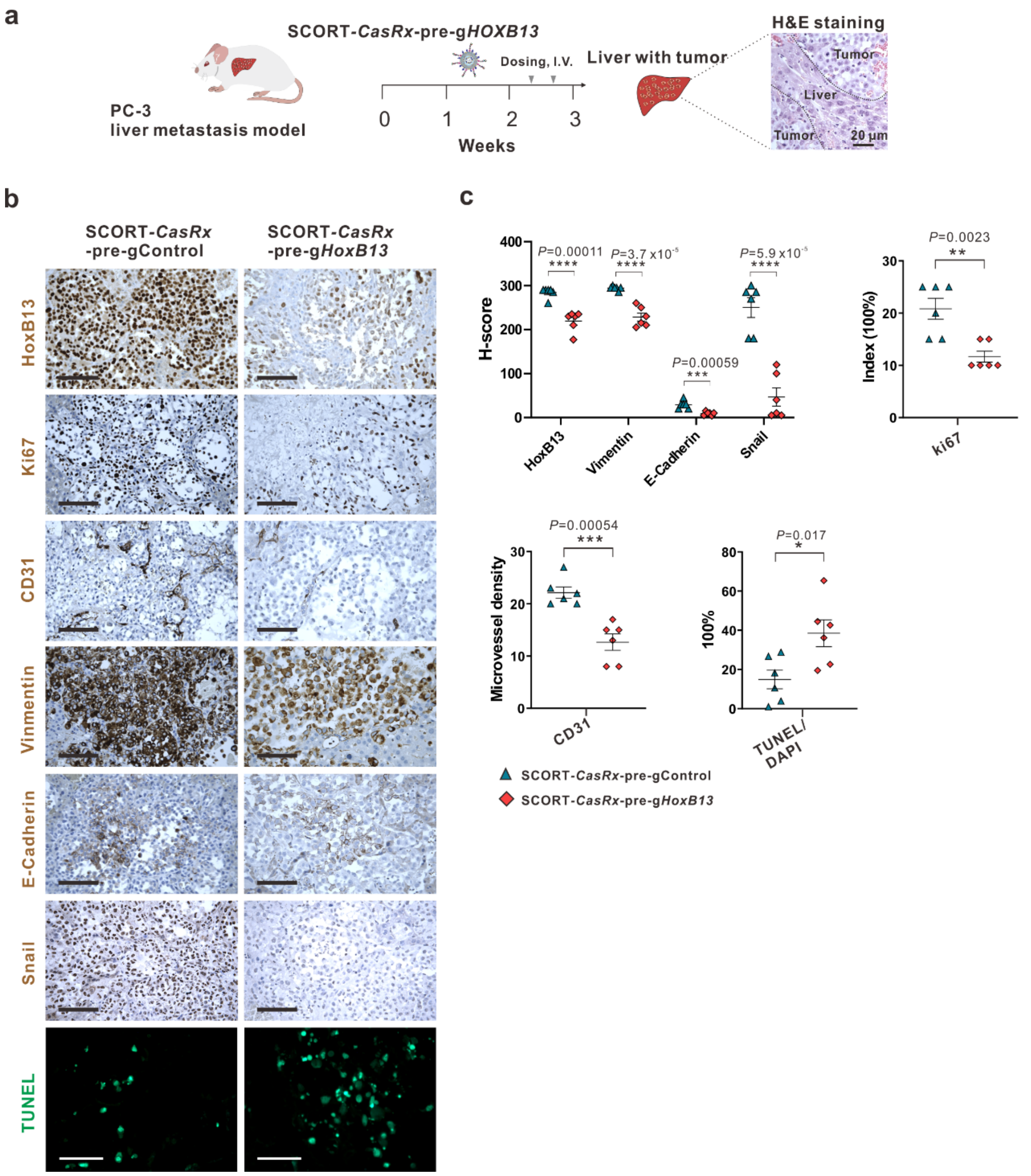

Figure S11. Direct cellular outcomes of SCORT-*CasRx*-pre-g*HoxB13* treatment in the PC-3 liver metastasis mouse model. a, Schematic illustration of the experimental design. Two weeks after PC-3 cell injection, mice received two doses of either SCORT-*CasRx*-pre-gControl or SCORT-*CasRx*-pre-g*HoxB13* with a 3-day interval. Three days after the second dose, liver samples with metastatic tumors were subjected for analysis (n=6). A representative photomicrograph of an H&E-stained liver section was also shown. Scale bar: 20 µm. **b**, Representative immunostaining images. Scale bar: 50 µm. **c**, Quantification of the immunostaining and TUNEL assay: H-scores for HoxB13, Vimentin, E-Cadherin and Snail; index for Ki67 and microvessel density indicated by CD31. Data are presented as mean ± SEM. *P* values were calculated by two tailed Student's *t*-tests, **P*<0.05, ***P*<0.01, ****P*<0.001, *****P*<0.0001.

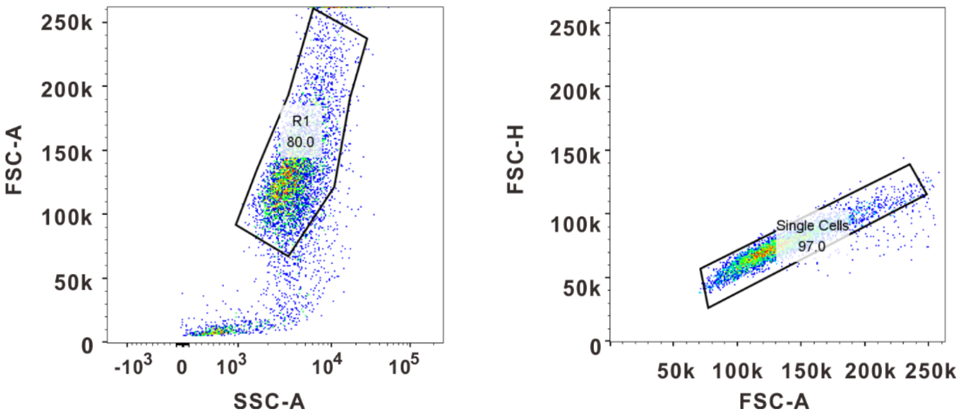

Figure S12. The gating strategy employed in flow cytometry experiments. Cell debris and doublets were excluded by forward scatter (FSC) and side scatter (SSC) parameters, and this gating strategy was consistently applied to all samples.

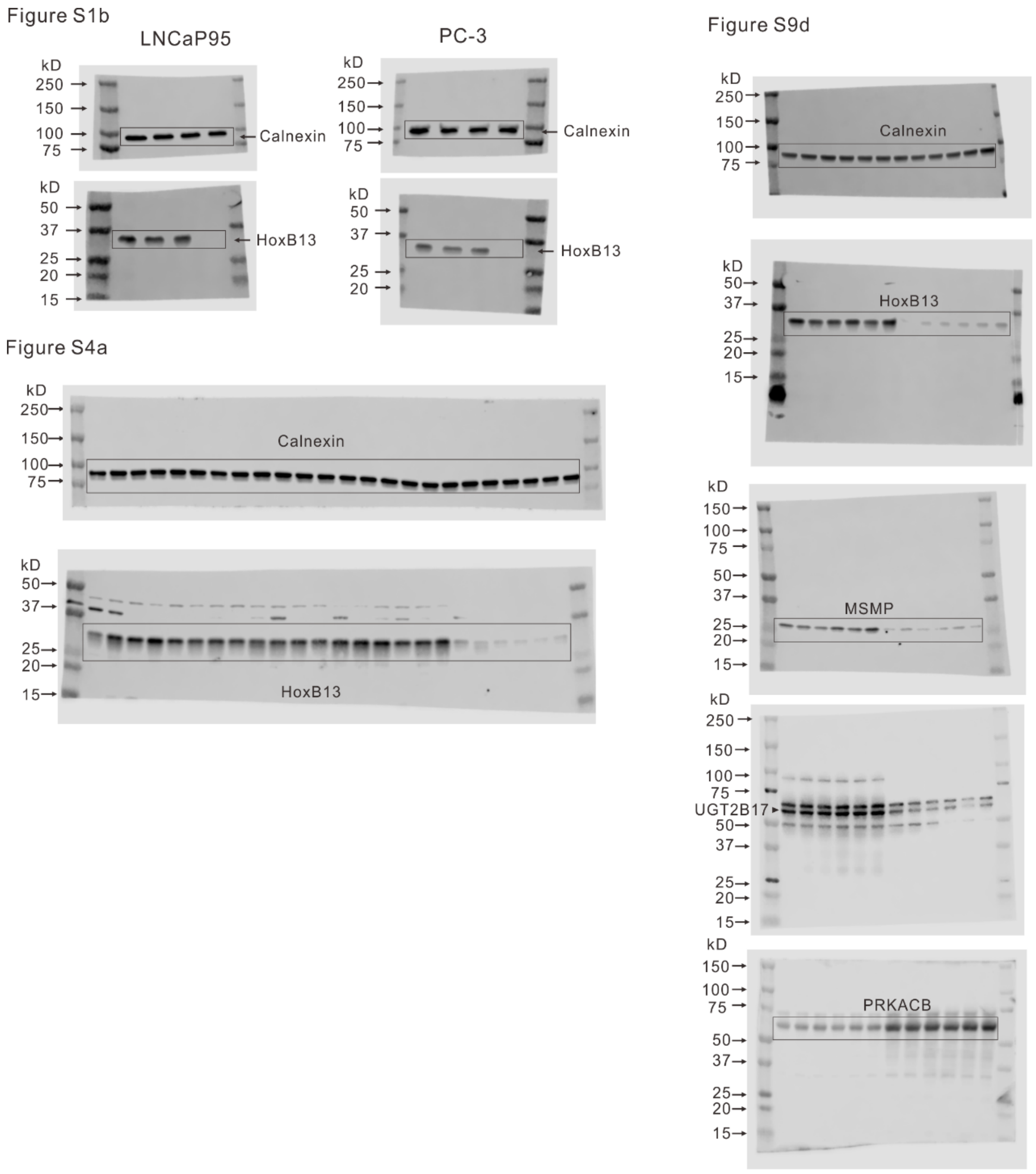

Figure S13. Full sets of unmodified raw Western blots for all analyzed proteins.

Extended Data Table 1 | Sequences for pre-gRNA screening and oligos with modification

Name	Sequence (5' to 3')
HoxB13 pre-gControl	TCACCAGAAGCGTACCATACTCACGAACAG
HoxB13 pre-gRNA-1	TCCCGGATAGAAGGCAAACTCAGTGGGGCG
HoxB13 pre-gRNA-2	AGATCTCTTAAGGGGTAGCGCTGTTCTTCA
HoxB13 pre-gRNA-3	AGGGGGCATAGTTGACAGCAGGCATCAGCG
HoxB13 pre-gRNA-4	CAAAAGGGACCTGGTGGGTTCTGTTCTCCC
pre- g*HoxB13*	__C__*__A__*__A__*GUA__A__*__A__*__C__*CCCUACCAACUGGUCGGGGUUUGA<br>AAC__C__AAAAGGGA__CCU__GG__U__GGG__UUCU__GUUCUCCCCAAG<br>UAAACCCCUACCAACUGGUCGGGGUUUG__A__*__A__*__A__*C
pre-gControl	__C__*__A__*__A__*GUA__A__*__A__*__C__*CCCUACCAACUGGUCGGGGUUUGA<br>AAC__UC__A__CC__AGAAG__C__G__U__A__CC__A__U__A__CUC__ACGAACAGCAAG<br>UAAACCCCUACCAACUGGUCGGGGUUUG__A__*__A__*__A__*C
	__N__: 2'OMe modification; *: phosphorothioate
Apt-E3	{SHC6.}GG__CUUUC__GGG__CUUUC__GG__C__AA__C__A__UC__AG__CCCCUC__AG__CC__
Apt-Control	{SHC6.}GG__C__G__U__AG__U__GA__UU__A__U__GAA__UC__G__U__G__U__G__CU__AA__U__A__C__A__C__G__CC__
	__N__: 2'Fluoro; {SHC6.} 5' C6 Thiol Linker

Extended Data Table 2 | Primer sequence

Name	Sequence (5' to 3')
HoxB13- F	ACAGAACCCACCAGGTCCCTTT
HoxB13-R	TACGGAATGCGTTTCTTGCGGC
18S-rRNA- F	GTAACCCGTTGAACCCCATT
18S-rRNA- R	CCATCCAATCGGTAGTAGCG
SNAI1-F	TCGGAAGCCTAACTACAGCGA
SNAI1-R	AGATGAGCATTGGCAGCGAG
CDH1-F	CGAGAGCTACACGTTCACGG
CDH1-R	GGGTGTCGAGGGAAAAATAGG
MSMP-F	ATGGCCCTAAGGATGCTCTG
MSMP-R	TGGAGGAGTAGAGACATCACCA
SKP2-F	ATGCCCCAATCTTGTCCATCT
SKP2-R	CACCGACTGAGTGATAGGTGT
MCM3-F	TCAGAGAGATTACCTGGACTTCC
MCM3-R	TCAGCCGGTATTGGTTGTCAC
CCNB1-F	AATAAGGCGAAGATCAACATGGC
CCNB1-R	TTTGTTACCAATGTCCCCAAGAG
MYC-F	GGCTCCTGGCAAAAGGTCA
MYC-R	CTGCGTAGTTGTGCTGATGT
POLD2-F	CCATCAGCCAACAATGCCAC
POLD2-R	CTAGCCGGAAGGGTTGTGA
NEIL3-F	TCTCCTGTTTTGGAAGTGCAG
NEIL3-R	CATTAGCACATCACCTAGCATCC
NTHL1-F	CGCGGAAAGCACAGAGACT
NTHL1-R	CTCATGGCACGGATGTTGAC
LIG1-F	GAAGGAGGCATCCAATAGCAG
LIG1-R	ACTCTCGGACACCACTCCATT
UGT2B7-F	GATCCCAACAACTCATCCGCT
UGT2B7-R	CAGCAGCTCACTACAGGGAA
UGT2B15-F	CCAACCAATGAAGCCCCTG
UGT2B15-R	GTTGTGAGCTGCGACTCGAA
UGT2B28-F	TCTGCTGATACATCTCGGTTGT
UGT2B28-R	GAGTGAATGCGTCATTGGGAT
UGT2B17-F	GCGACAAAACGTCTTTTCACAAC
UGT2B17-R	GCTCAGTAACTTTTGTGTGGGG
UGT2B11-F	AGCTCATATTGAAATTTGTTGCACT
UGT2B11-R	AGAATCCTGTTCAAAATGAAGCCA
CLDN4-F	TGGGGCTACAGGTAATGGG
CLDN4-R	GGTCTGCGAGGTGACAATGTT
AMOTL2-F	GCTCGTTGAGTGAACGGCT
AMOTL2-R	CATGAGCTAGTACAACATGAGGG
PRKACB-F	CCATGCACGGTTCTATGCAG
PRKACB-R	GTCTGTGACCTGGATATAGCCTT
ATG9B-F	CCCCTCATACAAGAAGCTCCC
ATG9B-R	TGCAGGTTGAGCCTGTGTTG
ZNF793-F	TCAGTGGGTTATGAAGGCACC
ZNF793-R	GCATGTCTTGCCGTCTTTTCC
ZNF727-F	TATTGCTGGAGCACGACATAAAC
ZNF727-R	GATTGAGCACAACCCAAAAGC

Name	Sequence (5' to 3')
ZNF737-F	GCTTTAAGCTATCCCACTATGCT
ZNF737-R	TGTTGGGTTTCTTTCCCGCA
PLK2-F	CTACGCCGCAAAAATTATTCCTC
PLK2-R	TCTTTGTCCTCGAAGTAGTGGT
GADD45A-F	GAGAGCAGAAGACCGAAAGGA
GADD45A-R	CACAACACCACGTTATCGGG
AXIN2-F	CAACACCAGGCGGAACGAA
AXIN2-R	GCCCAATAAGGAGTGTAAGGACT
CTSW-F	AGATCCAGTTCAACCGGAGTT
CTSW-R	TGTGAGGTCACTGAATGGAGT
FCAMR-F	GAGAAGTGGACTACTCCAGGC
FCAMR-R	GCGAAAGAAGAACCTTGTAGCAG
DHX38-F	TCCCTGAAACGGAGAGAGC
DHX38-R	CCCGAGCAGACCGATAATGT
MEX3B-F	AAGAGCGTGAACATGACCGAG
MEX3B-R	CGCTTTGATTTTACAACCTTGCC
DDX23-F	GCGATCACGGACTCCTGAC
DDX23-R	GAACGATGCCGCTTTCTATCT
VEGFA-F	AGGGCAGAATCATCACGAAGT
VEGFA-R	AGGGTCTCGATTGGATGGCA
CERS3-F	GGATCACGATGGACTCGTCTT
CERS3-R	GGTTGCCTTGTGGAATGTTTGA
TK1-F	GGGCAGATCCAGGTGATTCTC
TK1-R	TGTAGCGAGTGTCTTTGGCATA
Tnf -F	CAGGCGGTGCCTATGTCTC
Tnf-R	CGATCACCCCGAAGTTCAGTAG
Il-6 -F	CTGCAAGAGACTTCCATCCAG
Il-6– R	AGTGGTATAGACAGGTCTGTTGG
IL-2-F	GCACCCACTTCAAGCTCCA
IL-2-R	AAATTTGAAGGTGAGCATCCTG
IFNα-F	TGCCCAGCAGATCAAGAAGG
IFNα-R	TCAGGGGAAATTCCTGCACC
IFNβ-F	CGTGGGAGATGTCCTCAACT
IFNβ-R	AGATCTCTGCTCGGACCACC
Isg15-F	CGATTTCCTGGTGTCCGTGA
Isg15-R	AGCCAGAACTGGTCTTCGTG
Cxcl10-F	CCAAGTGCTGCCGTCATTTTC
Cxcl10-R	TCCCTATGGCCCTCATTCTCA
Ccl5-F	TTTGCCTACCTCTCCCTCG
Ccl5-R	CGACTGCAAGATTGGAGCACT
Ccl2-F	TAAAAACCTGGATCGGAACCAAA
Ccl2-R	GCATTAGCTTCAGATTTACGGGT
